# Conservation of cortical crowding distance across individuals in human V4

**DOI:** 10.1101/2024.04.03.587977

**Published:** 2024-04-05

**Authors:** Jan W. Kurzawski, Brenda S. Qiu, Najib J. Majaj, Noah Benson, Denis G. Pelli, Jonathan Winawer

**Affiliations:** 1 Department of Psychology, New York University, New York, NY, USA; 2 Department of Psychology, University of Washington, Seattle, WA, USA; 3 Center for Neural Science, New York University, New York, NY, USA; 4 eScience Institute, University of Washington, Seattle, WA, USA

## Abstract

Crowding is the failure to recognize an object due to insufficient spacing, slowing daily tasks such as reading and search. Across 49 observers, we found large variations in psychophysical crowding distance and retinotopic map size. These measures covary, conserving a 1.4-mm *cortical crowding distance* (threshold object spacing on the cortical surface) in the human V4 map, but not V1–V3, linking the spacing limit of visual recognition to overall V4 size.

Visual recognition is limited by the size and spacing of objects^1^. The size limit is understood but the spacing limit is not. The size limit (acuity) is set by properties of the eye, including optical quality and the sampling density of the retinal cell mosaics^2^. However, even when image features are much larger than the acuity limit, recognition fails if objects are insufficiently spaced (crowding). Crowding limits performance of important everyday activities, including reading and search^3,4^. The biological basis of crowding is unknown except that it must be cortical, because crowding persists when target and clutter are presented separately to the two eyes^5^.

Crowding is quantified by distance, the minimum center-to-center spacing for target recognition. There are large individual differences in crowding distance even among normally sighted young adults^6–9^. Here, we use this variability to investigate the neural basis of crowding. We conjecture that crowding results from limited neural resources at some processing stage, similar to how acuity is limited by retinal cell density. Retinotopic maps in the human cortex are obvious candidates to mediate crowding. We ask whether individual differences in surface area of one or more retinotopic maps explain crowding distance variability across observers.

We do so by testing a cortical constancy conjecture, as follows. In each of 49 observers, we measure the surface area of retinotopic maps V1 to V4 with fMRI and the crowding distance with a psychophysical letter identification task. From the crowding distance, we derive an estimate of *λ*, the number of letters that fit into the visual field without crowding (Figure 1A). The constancy conjecture is that variation in *λ* is entirely due to variation in surface area, *A*, of one or more maps, such that observers with larger maps can recognize more letters in their visual field (Figure 1B). We express this relationship as,

(1) λ=kA,

where *λ* and *A* are vectors with an element per observer, and *k* is a scalar in units of letters per mm^2^ of cortex. We can re-express this relationship in terms of the cortical crowding distance *c*, which measures spacing in mm on the cortex between target and flanker at threshold spacing:

(2) $$A=c^{2}\lambda ,$$

so

(3) $$c=\frac{1}{\sqrt{.k}},$$

Prior work observed that radial crowding distance, in degrees of visual angle, increases linearly with eccentricity (the Bouma law), and that radial cortical magnification, measured in mm/deg, decreases inversely with eccentricity, such that the product of the two functions is roughly constant^10^. This amounts to conservation across eccentricity of the threshold target-to-flanker distance on the cortical surface. This conservation applies equally to any retinotopic map (including V1 to V4) where cortical magnification decreases inversely with eccentricity, without favoring any map for association with psychophysical crowding^10^. While conservation across eccentricity has not been shown to distinguish visual maps, conservation across individuals might. Indeed, size variation in retinotopic maps across individuals is largely independent, meaning that the size of one map (e.g., V1) only weakly predicts the size of others (e.g., V4)^11^. Here we test each retinotopic map for conservation of crowding distance across individuals, with reason to expect that it cannot be conserved in multiple maps.

Consider two actual observers, one with a small and one with a large *λ* (Figure 2A). *λ* is calculated from crowding distances measured at 2 eccentricities at 4 meridians (Supplementary Figure 1). The *λ* of Observer 1 is more than twice that of Observer 2. The surface area of V4 in Observer 1 is also more than twice that of Observer 2, whereas the surface areas of V1 to V3 are similar (Figure 2B).

We then assessed the reliability of measured individual differences across our population of observers. We estimated *λ* twice per observer and found a high correlation between sessions (*r* = 0.94). Similarly, we measured *A* twice by having two researchers independently draw the retinotopic map boundaries and found high agreement in surface area between the two researchers (*r* = 0.94, 0.88, 0.74, 0.73 for V1 to V4, respectively). We assume that additional uncertainty in *A* from scan-to-scan variability is negligible compared to differences in how researchers use heuristics to draw map boundaries. Retinotopic mapping parameters in the early visual cortex are stable across scans^12,13^. The large-scale features of the retinotopic data, such as the V1/V2 polar angle reversals, are even more stable than individual voxel data, since much of the random measurement noise in each voxel tends to cancel out.

To compare crowding and surface area, for each observer we plot one point at *A* vs *λ* for V1 to V4 (Figure 3A). If the cortical crowding distance is the same in each observer, i.e. if a map conserves crowding distance, then *λ* will scale with *A*. Only in V4 do we find high correlation between *λ* and *A* (*r* = 0.65). In V1 to V3, there is no relationship between *λ* and *A*, indicated by the circular covariance ellipses in the scatterplots and the zero-correlation coefficient (Figure 3B). We then explicitly tested the conservation hypothesis, by quantifying the variance in *λ* explained by a scale factor of *A*. The variance explained is high in V4 (0.43) and zero in V1 to V3 (Figure 3C).

The slope of the conservation line allows us to estimate the spacing between letters on the V4 map. The slope, *k*, is 0.54 [CI 0.52 – 0.57] letters per mm^2^ from which we derive the cortical crowding distance, *c*, 1.4 mm [CI 1.33 – 1.52]. This single number, 1.4 mm, is the required letter-to-letter spacing across people, despite very different map sizes and crowding distances.

*Why is cortical crowding distance conserved?* Our main finding is that despite large individual differences in psychophysical crowding distance and map size, crowding distance is conserved on the V4 map but not on V1 to V3. Both crowding and V4 are thought to be associated with mid-level visual processes^14,15^. We speculate that the 1.4 mm crowding distance is conserved because there is a computational unit composed of a fixed number of V4 neurons needed to isolate and recognize an object. A fixed neural count implies a fixed area, which we estimate at *A*/*λ*=2 mm^2^ of cortex. This is analogous to a V1 hypercolumn analyzing local scale and orientation, with an area of 1.5 mm^2^ in human^16^. In seeking a link between behavior and brain measures, we emphasized anatomy (map size) rather than physiology (neural response properties). The measured map size is invariant to task, stimulus, and attentional state (see Extended Figure 9 in ref [^17^]). Previous physiological methods have yielded ambiguous results linking crowding with various visual maps, including V1^18^, V2^19,20^, V3^19^, and V4^21,22^ (Supplementary Table 1). The ambiguity is partly due to the gradual degradation of target-related neural responses in successive processing stages. Hence the identification of a bottleneck relies on the choice of stimulus and assumptions about how much degradation matters for behavior. This is further complicated by the fact that the physiological studies cannot sample entire brain areas, and that the comparison to behavior is indirect – relying on group-averaged human psychophysics. In contrast, the individual-differences approach here, measuring both crowding and maps in the same 49 individuals, shows a sharp divide, with no relationship between psychophysics and map size in V1 to V3, and a strong relationship in V4.

We used an individual differences approach to uncover the biological basis of crowding. These individual variations are important in their own right. Extreme individual variation becomes a disorder, as in dyslexia and dyscalculia, which are associated with unusually large crowding distances^23,24^. Our discovery that the size of the V4 map predicts crowding might help understand these disorders.

## Methods

### Participants

Fifty participants (32 females, 18 males, mean age 27 years) were recruited from New York University. All participants had normal or corrected-to-normal vision. The participants completed one fMRI session to measure retinotopic maps (45 min) and two behavioral sessions to measure crowding distance (30 min each). One participant was dropped from the analysis due to incomplete fMRI data which did not allow for reliable estimation of retinotopic maps. All participants provided written informed consent and approved the public release of anonymized data. The experiment was conducted under the Declaration of Helsinki and was approved by the NYU ethics committee on activities involving human participants.

### Pilot study

We conducted a pilot study with 26 participants and reported the results in a conference abstract^1^. The results reported here are independent and highly consistent with the pilot study.

### fMRI stimulus display

Participants viewed a retinotopic mapping stimulus in the MRI scanner using a ProPixx DLP LED projector (VPixx Technologies Inc., Saint-Bruno-de-Montarville, QC, Canada). The stimulus image was projected onto an acrylic back-projection screen (60 cm × 36.2 cm) in the scanner bore. The projected image had a resolution of 1920 × 1080 and a refresh rate of 60 Hz. The display luminance was 500 cd/m^2^. Participants viewed the screen at 83.5 cm (from eyes to the screen) using an angled mirror that was mounted on the head coil.

### fMRI Stimuli

The mapping stimulus was generated in MATLAB 2017a and was presented using the Psychophysics Toolbox v3^2^ and custom vistadisp software (https://github.com/WinawerLab/vistadisp) on an iMac computer. For 27 participants, stimulus images were shown within a sweeping bar aperture, identical to the stimulus in Himmelberg et al^3^. For 33 participants, the stimulus image patterns were shown within two types of apertures in alternative scans, a sweeping bar and a wedge+ring. These stimulus apertures were similar to the ones used in the NSD dataset^4^. Unlike NSD, our stimuli changed location in discrete steps and had a larger maximum eccentricity. In short, there were six runs of retinotopic mapping, each lasting 5 minutes (300 TRs of 1 sec). Each aperture type was shown three times in an interleaved order. The carrier image pattern was presented with a 3 Hz temporal frequency and was composed of colorful objects, faces, and scenes at multiple scales. These images were superimposed on an achromatic pink-noise (1/f) background^5^. The stimulus image pattern was windowed within a circular aperture (12.4° of maximum radius). The apertures were superimposed on a polar fixation grid placed upon a uniform gray background, with a red or green dot at the center (3 pixels, or 0.07°). Participants completed a fixation task to ensure that they were maintaining central fixation and remained alert throughout the scan. Participants were required to respond, via button press, when the central fixation dot changed from green to red, or vice versa.

### Data acquisition and preprocessing

Structural and functional data were acquired on a 3T Siemens MAGNETOM Prisma MRI scanner (Siemens Medical Solutions, Erlangen, Germany) at the Center for Brain Imaging at NYU. Both the structural and functional images were acquired using a Siemens 64-channel head coil. MPRAGE anatomical images were acquired for each participant (TR, 2400 ms; TE, 2.4 ms; voxel size, 0.8mm^3^ isotropic; flip angle, 8°), and were auto-aligned to a template to ensure a similar slice prescription for all participants. Additionally, two distortion maps were acquired to correct susceptibility distortions in the functional images: one spin-echo image with anterior-posterior (AP) and one with posterior-anterior (PA) phase encoding. Anatomical and functional preprocessing was performed using fMRIPrep v.23.1.2^6^. For each participant and each run, fMRIprep produced a BOLD time series on the participant’s native freesurfer surface.

### Population receptive field model

We first averaged the time-series data from the three repetitions of bar aperture and three repetitions of wedge and ring apertures resulting in two functional time series. For the 27 participants who saw images only through a bar aperture, we averaged all 6 runs. We used these average data to fit the pRF model. The analysis was conducted using vistasoft (https://github.com/WinawerLab/prfVista). A pRF is modeled as a circular 2D-Gaussian, as described in Dumoulin and Wandell^7^. The Gaussian is parameterized by values at each vertex for x, y, and σ. The x and y parameters specify the center position of the 2D-Gaussian in the visual field. The σ parameter, the standard deviation of the 2D-Gaussian, specifies the size of the receptive field. The 2D-Gaussian is multiplied pointwise by the stimulus contrast aperture (apertures were prepared at a resolution of 101 pixels × 101 pixels), and then convolved with a hemodynamic response function (HRF) to predict the BOLD percent signal change. The HRF is parameterized by 5 values, describing a difference of two gamma functions, as used previously^7^. The HRF was assumed to be the same across vertices within a participant but differed among participants. We use a two-stage coarse-to-fine approach described in detail by Dumoulin and Wandell^7^, and the addition of the HRF fit is described in detail by Harvey and Dumoulin^8^

### Defining the size of visual areas (V1–V4)

Two authors, JWK and BSQ, independently defined ROIs (V1–V4) by hand using neuropythy (https://github.com/noahbenson/neuropythy)^9^. To define visual maps, researchers followed the heuristics of Wandell^10^ for V1–V3, and of Winawer and Witthoft^11^ for V4. To match the maximum eccentricity of the psychophysical stimuli we restricted all maps to a maximum eccentricity of 10 deg. The size of each map was calculated as the sum of the surface areas of all vertices included in the map on the mid-gray cortical meshes, defined by FreeSurfer as the surfaces hallway between the meshes at the gray/white interface and the pial/gray interface. Finally, each map surface area was summed across hemispheres.

### Measuring crowding distance

Data were acquired with the CriticalSpacing software^12^, allowing for reliable and relatively fast measurement of crowding distances. Our crowding database consists of measurements of crowding distance with the Sloan font with radial flankers. We measured crowding at 8 different peripheral locations in the visual field: 2 eccentricities (5, 10 deg) along the four cardinal meridians (upper, lower, left and right). Each observer participated in two crowding sessions (each lasting about 40 min). Sessions were separated at least by a day with a maximum of five days. In session one first block showed stimuli at 5 deg and the second showed stimuli at 10 deg. In the second session the order was reversed.

### Crowding stimulus display

Each testing session was completed on an Apple iMac 27” with an external monitor for stimulus presentation. Screen resolution was 5120 × 2880. Apple iMac has AMD graphics for optimal compatibility with the Psychtoolbox imaging software. The Systems Preference: Displays: Brightness slider was set (by calling MacDisplaySettings.m in the Psychtoolbox) to 0.86 (range 0 to 1) to achieve a white background luminance of about 250 cd/m^2^. The observer viewed the screen binocularly at 40 cm distance from the screen. Stimuli were rendered in MATLAB 2021 using the Psychtoolbox^2^

#### Crowding stimuli

To measure crowding we show a trigram of three letters. For each trial, the three letters are drawn randomly, without replacement, from the 9 letters. Letters are rendered as black in the Sloan font, presented on a uniform white background of about 250 cd/m^2^. For the Sloan font we use the letters “DHKNORSVZ” as possible targets and flankers. For crowding, each trigram is always arranged radially. Each testing session is about 30 minutes, measuring crowding for targets at two eccentricities, 5° and 10°. The eccentricities are measured in separate blocks of about 15 min each. Each block measures crowding thresholds at the four cardinal directions, with four interleaved staircases. Fixation is displayed until the observer presses a key to initiate the trial. Then the letter trigram appears on the screen for 150 ms and the computer waits for the observer to identify the middle letter by choosing the letter using a mouse click. Letter choices appear on the screen after stimulus offset. Observers are instructed to return their eyes to fixation before clicking their response. The computer waits indefinitely for each response. The next trial began 300 ms after the observer’s response, only if the observer was fixating in the center for at least 250 ms.

### Eye tracking

For all thresholds, we controlled for eye movements. To measure fixation, we used an Eyelink 1000 eye tracker (SR Research, Ottawa, Ontario, Canada) with a sampling rate of 1000 Hz. To allow for short viewing distance (40 cm) we used their Tower mount setup with a 25 mm lens mounted on the Eyelink camera. We allowed participants to move their eyes within a 1.5-degree radius from fixation. We checked eye position during the stimulus presentation. If during the stimulus presentation, observers moved their eyes more than 1.5 deg away from the fixation, the fixation cross turned red, and observers were asked to repeat the trial. Each threshold was estimated based on 35 trials with correct fixation. On average, 10% of trials had to be repeated.

### Estimating crowding distance

To estimate the crowding distance we used the procedure of Pelli^12^. Letter spacing is controlled by QUEST. Spacing scales with letter size, maintaining a fixed ratio of 1.4:1. We set the guessing rate parameter gamma to the reciprocal of the characters in the test alphabet for that font, usually 9. We set the “finger error” probability delta to 1%, so that QUEST can better cope with an occasional reporting mistake. At the end of each block, QUEST estimates the threshold (crowding distance). We devote 35 trials to measure the threshold for a condition. In each block we randomly interleaved meridian locations, to get a threshold for each condition, while keeping the observer uncertain as to which condition is present on each trial.

### Calculating the Bouma factor *b*

For each participant we collected 16 crowding thresholds (2 eccentricities × 4 cardinal meridians × 2 sessions). For each session crowding distances were converted to Bouma factors by dividing them by corresponding target eccentricity. Next, we estimated one Bouma factor for each session by taking the geometric mean across all 8 visual field locations. The final estimate for each observer was calculated as the geometric mean of Bouma factor from session 1 and 2.

### Calculating number of letters for uncrowded arrays

We derived *λ*, the number of letters that fit into the uncrowded visual field, from Bouma’s law. In 1-D, Bouma’s law is

(4) $$s_{r}=\left(\varphi_{0}+\varphi \right)b,$$

where *s*_*r*_ is the radial crowding distance in deg, *φ* is the target eccentricity in deg, and *φ*_0_ and *b* are fitted constants. The Bouma law can be extended to include flanker orientation, such that

(5) $$s_{t}=\hat{s_{r}}/\alpha ,$$

where *s*_*t*_ is the crowding distance with tangentially oriented flankers, and *α* is the ratio of radial-to-tangential crowding distance.

When letters are packed as tightly as possible into the uncrowded visual field (i.e., spaced everywhere by the local crowding distance), the local density in letters per deg^2^, is the product of the radial and tangential crowding distances:

(6) $${\left(s_{r}s_{t}\right)}^{-1},$$

The integral of this expression, in polar coordinates, is *λ*, the number of letters in the uncrowded visual field:

(7) $$\lambda =\int_{0}^{\varphi_{\text{max}}}\int_{0}^{2\pi \varphi }/\left(s_{r}s_{t}\right)d\theta d\varphi ,$$

where *φ* is the eccentricity, *φ*_*max*_ is the maximum extent of the visual field (in deg), θ is the polar angle, and *s*_*r*_ and *s*_*t*_ are the radial and tangential crowding distance. We measured *λ* for *φ*_*max*_ = 10, and we assumed the values of *α* and *φ*_0_ from our prior study (Kurzawski et al. 2023) as 2 and 0.24, respectively. With these settings, the integral above reduces to

(8) $$\lambda =34.9/b^{2},$$

We conducted a sensitivity analysis of *λ* by varying the settings of *α* and *φ*_0_ (Supplementary analysis 1).

### Relationship between *A and λ*

**Figure 3.**
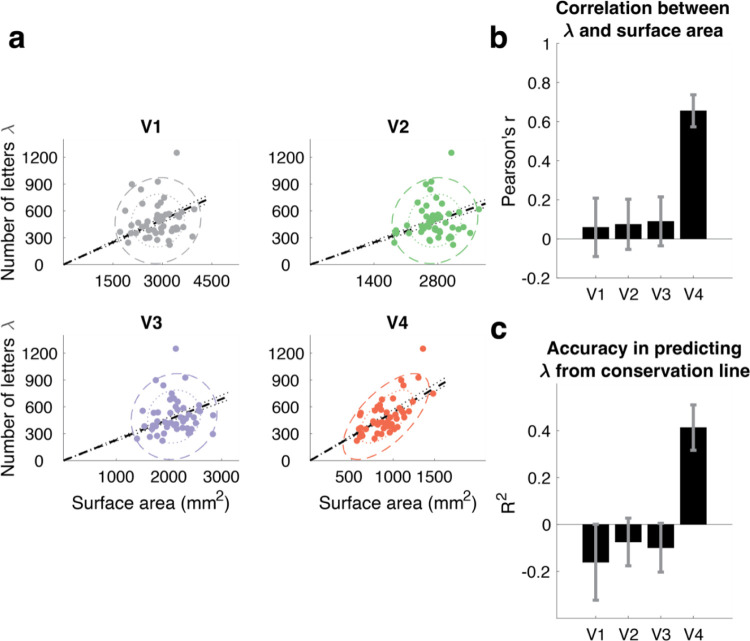
A test of conservation of cortical crowding distance in 4 retinotopic maps. **A**. Each panel plots one point per observer as *λ*, the number of letters that fit into the uncrowded visual field (central 10 deg bilaterally) vs *A*, the surface area of a visual map (summed across hemispheres), for 49 observers. Black outlines around two dots in each panel show data for the two observers from Figure 2. The colored ellipses are the 1-SD and 2-SD contours from the covariance ellipses. The black dashed lines are the fits by regression with one parameter, slope, i.e., the prediction assuming conservation. The dotted black line shows 68th percentile of the conservation fit. The slope of this line has units of letters/mm^2^ and is equal to 1/c^2^, where c is the cortical crowding distance. In V4, but not V1 to V3, the two-parameter fit of the data closely agrees with conservation. **B**. The correlation between *λ* and *A* for V1–V4. **C**. The *R*^2^ value is the coefficient of determination, the amount of variance in *λ* explained by assuming conservation (one-parameter fit). To estimate confidence intervals in Panels A (conservation line), B, and C, we bootstrapped across observers (n = 10,000).

We quantified the relationship between *A*, cortical map surface area, and *λ*, number of letters in the uncrowded visual field, in two ways. First, we computed the correlation (Pearson’s *r*) and the covariance between the two variables. These calculations treat the two variables symmetrically. We visualize the covariance ellipses at 1-SD and 2-SD, scaling the x and y axes so that one standard deviation of each variable is the same length (Figure 3A). This ensures that the covariance ellipses are circular when the correlation is 0. Second, to explicitly test for conservation, we fit a linear model to *A* (independent variable) and *λ* (dependent variable), with the intercept set to 0, and asked how much of the variance in *λ* is explained (Figure 3C). Note that the variance explained can be negative, because a line through the origin may be a worse predictor (larger residuals in *λ*) than just assuming the mean (a horizontal line). For both the correlation coefficient and the variance explained, 68% confidence intervals were calculated by bootstrapping participants with replacement (n = 10000).

## Supplementary Material

1

## Figures and Tables

**Figure 1.**
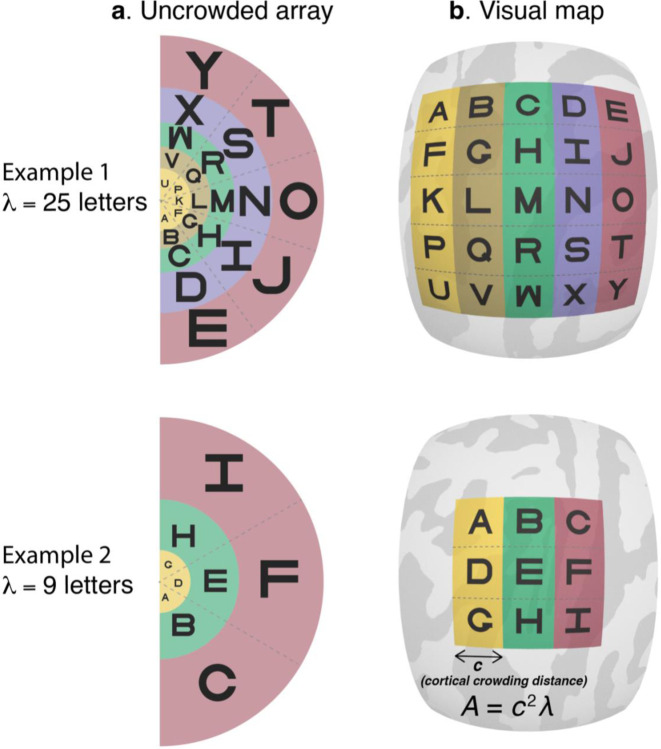
Conservation of cortical crowding distance. The left column shows two hypothetical examples of uncrowded visual fields. In each case, the letters are spaced at the minimum distance necessary for successful recognition. The lower spacing in the upper row indicates that this hypothetical observer has a small crowding distance, and hence can fit more letters into the uncrowded visual field (*λ* = 25 vs *λ* = 9). The right column shows the representation of these two visual fields in retinotopic maps on the cortical surface, assuming cortical conservation of crowding. According to this conjecture, the greater *λ* for the first observer is explained by a larger cortical map. The center-to-center letter spacing on the cortex in mm is *c*, the same for both observers. The surface area of the map, *A*, is *c*^2^*λ*, the product of the area per letter and number of letters. Hence the conservation conjecture predicts that the number of letters in the uncrowded field is proportional to surface area.

**Figure 2.**
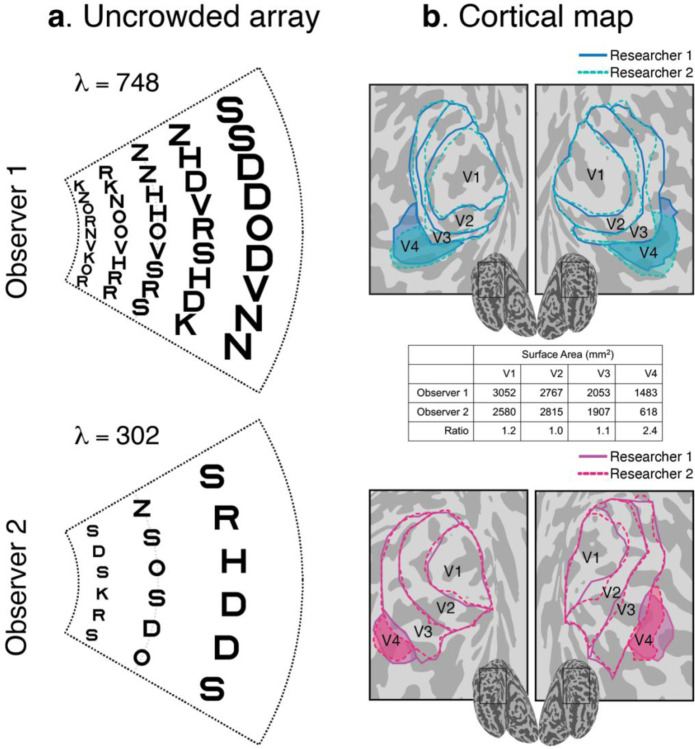
Individual differences in map size and *λ*. A) For each observer, we show a portion of the visual field with letters spaced according to their Bouma factors. Observer 1 has a small Bouma factor (0.21 radially) and can individuate many letters, *λ* = 748. Observer 2 has a large Bouma factor (0.34 radially) and small *λ* = 302. The portion of the visual field shown is 2.5° to 10° eccentricity, ±25° polar angle. The *λ* values only count letters within 10° eccentricity. B) Retinotopic map boundaries (V1 to 1) from both hemispheres of the same two observers. Visual maps were independently drawn by two researchers (dashed and solid lines). The numbers in the table indicate the surface area, *A*, in mm^2^ of the bilateral retinotopic maps, averaged across the two researchers. Observer 1 has a much larger hV4 than Observer 2. Retinotopic maps for the two observers are shown in the Supplementary Figure 2.

## Data Availability

Code to produce figures and raw crowding data together with MRI surface files are deposited on GitHub. fMRI dataset in BIDS format will be available on OpenNeuro upon publication.

## References

[1] Song S., Levi D. M. & Pelli D. G. A double dissociation of the acuity and crowding limits to letter identification, and the promise of improved visual screening. Journal of Vision 14, 3, doi:10.1167/14.5.3 (2014). PMC4021854 24799622

[2] Geisler W. S. Physical limits of acuity and hyperacuity. J Opt Soc Am A 1, 775–782, doi:10.1364/josaa.1.000775 (1984). 6747742

[3] Pelli D. G. Crowding and eccentricity determine reading rate. Journal of Vision 7, 20, doi:10.1167/7.2.20 (2007). 18217835

[4] Rosenholtz R., Huang J., Raj A., Balas B. J. & Ilie L. A summary statistic representation in peripheral vision explains visual search. Journal of Vision 12, doi:10.1167/12.4.14 (2012). PMC4032502 22523401

[5] Kwon M., Bao P., Millin R. & Tjan B. S. Radial-tangential anisotropy of crowding in the early visual areas. Journal of Neurophysiology 112, 2413–2422, doi:10.1152/jn.00476.2014 (2014). 25122703 PMC4233277

[6] Kurzawski J. W. The Bouma law accounts for crowding in 50 observers. Journal of Vision 23, 6, doi:10.1167/jov.23.8.6 (2023). PMC10408772 37540179

[7] Kurzawski J. W. EasyEyes — A new method for accurate fixation in online vision testing. Front. Hum. Neurosci. 17, doi:10.3389/fnhum.2023.1255465 (2023). PMC10718086 38094145

[8] Toet A. & Levi D. M. The two-dimensional shape of spatial interaction zones in the parafovea. Vision Res 32, 1349–1357, doi:10.1016/0042-6989(92)90227-a (1992). 1455707

[9] Veríssimo I. S., Hölsken S. & Olivers C. N. L. Individual differences in crowding predict visual search performance. Journal of Vision 21, 29, doi:10.1167/jov.21.5.29 (2021). 34038508 PMC8164367

[10] Pelli D. G. Crowding: a cortical constraint on object recognition. Curr Opin Neurobiol 18, 445–451, doi:10.1016/j.conb.2008.09.008 (2008). 18835355 PMC3624758

[11] Benson N. C. Variability of the Surface Area of the V1, V2, and V3 Maps in a Large Sample of Human Observers. J. Neurosci. 42, 8629–8646, doi:10.1523/JNEUROSCI.0690-21.2022 (2022). 36180226 PMC9671582

[12] Himmelberg M. M. Cross-dataset reproducibility of human retinotopic maps. NeuroImage 244, 118609, doi:10.1016/j.neuroimage.2021.118609 (2021). 34582948 PMC8560578

[13] van Dijk J. A., de Haas B., Moutsiana C. & Schwarzkopf D. S. Intersession reliability of population receptive field estimates. NeuroImage 143, 293–303, doi:10.1016/j.neuroimage.2016.09.013 (2016). 27620984 PMC5139984

[14] Anderson B. L. Mid-level vision. Curr Biol 30, R105–R109, doi:10.1016/j.cub.2019.11.088 (2020). 32017875

[15] Pelli D. G. & Tillman K. A. The uncrowded window of object recognition. Nat Neurosci 11, 1129–1135, doi:10.1038/nn.2187 (2008). 18828191 PMC2772078

[16] Garcia-Marin V., Kelly J. G. & Hawken M. J. Neuronal composition of processing modules in human V1: laminar density for neuronal and non-neuronal populations and a comparison with macaque. Cereb Cortex 34, bhad512, doi:10.1093/cercor/bhad512 (2024). 38183210 PMC10839852

[17] Allen E. J. A massive 7T fMRI dataset to bridge cognitive neuroscience and artificial intelligence. Nat Neurosci 25, 116–126, doi:10.1038/s41593-021-00962-x (2022). 34916659

[18] Millin R., Arman A. C., Chung S. T. L. & Tjan B. S. Visual Crowding in V1. Cereb Cortex 24, 3107–3115, doi:10.1093/cercor/bht159 (2014). 23833128 PMC4224237

[19] Bi T., Cai P., Zhou T. & Fang F. The effect of crowding on orientation-selective adaptation in human early visual cortex. Journal of Vision 9, 13.11–10, doi:10.1167/9.11.13 (2009). 20053076

[20] Freeman J. & Simoncelli E. P. Metamers of the ventral stream. Nat Neurosci 14, 1195–1201, doi:10.1038/nn.2889 (2011). 21841776 PMC3164938

[21] Henry C. A. & Kohn A. Feature representation under crowding in macaque V1 and V4 neuronal populations. Curr Biol 32, 5126–5137.e5123, doi:10.1016/j.cub.2022.10.049 (2022). 36379216 PMC9729449

[22] Motter B. C. Stimulus conflation and tuning selectivity in V4 neurons: a model of visual crowding. Journal of Vision 18, 15, doi:10.1167/18.1.15 (2018). PMC5783327 29362808

[23] Bertoni S., Franceschini S., Ronconi L., Gori S. & Facoetti A. Is excessive visual crowding causally linked to developmental dyslexia? Neuropsychologia 130, 107–117 (2019). 31077708 10.1016/j.neuropsychologia.2019.04.018

[24] Castaldi E., Turi M., Gassama S., Piazza M. & Eger E. Excessive visual crowding effects in developmental dyscalculia. Journal of Vision 20, 7, doi:10.1167/jov.20.8.7 (2020). PMC7438630 32756882

