# Conservation of cortical crowding distance across individuals in human V4

**DOI:** 10.1101/2024.04.03.587977

**Published:** 2024-04-29

**Authors:** Jan W. Kurzawski, Brenda S. Qiu, Najib J. Majaj, Noah Benson, Denis G. Pelli, Jonathan Winawer

**Affiliations:** 1 Department of Psychology, New York University, New York, NY, USA; 2 Department of Psychology, University of Washington, Seattle, WA, USA; 3 Center for Neural Science, New York University, New York, NY, USA; 4 eScience Institute, University of Washington, Seattle, WA, USA

## Abstract

Crowding is the failure to recognize an object due to insufficient spacing, which slows daily tasks such as reading and search. Across 49 observers, we found large variations in psychophysical crowding distance and retinotopic map size. These measures covary, conserving a 1.4-mm *cortical crowding distance* (threshold object spacing on the cortical surface) in the human V4 map, but not V1-V3. This links the spacing limit of visual recognition to overall V4 size.

Visual recognition is limited by the size and spacing of objects^1^. The size limit is understood but the spacing limit is not. The size limit (acuity) is set by properties of the eye, including optical quality and the sampling density of the retinal cell mosaics^2^. However, even when image features are much larger than the acuity limit, recognition fails if objects are insufficiently spaced (crowding). Crowding limits performance of important everyday activities, including reading and search^3,4^. The biological basis of crowding is unknown except that it must be cortical, because crowding persists when target and clutter are presented separately to the two eyes^5^.

“Crowding distance” is the minimum center-to-center spacing of objects needed for recognition. There are large individual differences in crowding distance even among normally sighted young adults^6–9^. Here, we use this variability to investigate the neural basis of crowding. We conjecture that crowding results from limited neural resources at some processing stage, similar to how acuity is limited by retinal cell density. Retinotopic maps in the human cortex are obvious candidates to mediate crowding. We ask whether individual differences in surface area of one or more retinotopic maps explain crowding distance variability across observers.

We do so by testing a cortical conservation conjecture, as follows. In each of 49 observers, we first psychophysically measure the crowding distance with a letter identification task, and then anatomically measure the surface area of retinotopic maps V1 to V4 with fMRI. Inspired by the letter acuity charts introduced by Anstis in 1974^10^, we calculate *λ*, the number of letters that fit into the visual field without crowding (Figure 1a). Conservation implies that variation in *λ* is entirely due to variation in surface area *A* of one or more maps, such that observers with larger maps can recognize more letters in their visual field (Figure 1b). We express this relationship as,

(1) λ=kA,

where *λ* (letters) and *A* (mm^2^) are vectors with an element per observer, and *k* is a scalar (letters per mm^2^ of cortex). We can re-express this relationship in terms of the cortical crowding distance *c*, which measures spacing in mm on the cortex between target and flanker at threshold spacing:

(2) $$A=\lambda c^{2},$$

where

(3) $$k=c^{-2}.$$

Prior work observed that radial crowding distance, in degrees of visual angle, increases linearly with eccentricity (the Bouma law), and that radial cortical magnification, measured in mm/deg, decreases inversely with eccentricity, such that the product of the two functions is roughly constant^11^ (but see Strasburger et al. 2022^12^). This amounts to conservation across eccentricity of the threshold target-to-flanker distance on the cortical surface. This conservation applies equally to any retinotopic map (including V1 to V4) where cortical magnification decreases inversely with eccentricity, without favoring any map for association with psychophysical crowding^11^. While conservation across eccentricity has not favored any of the V1 to V4 visual maps, conservation across individuals might. Indeed, across individuals, the sizes of retinotopic maps vary largely independently, meaning that the size of one map (e.g., V1) only weakly predicts the size of others (e.g., V4), with the exception of adjacent maps such as V1 and V2^13^. Here we test each retinotopic map for conservation of crowding distance across individuals, with reason to expect that it cannot be conserved in multiple maps.

Consider two actual observers, one with a large *λ* and another with a small *λ* (Figure 2a). *λ* is calculated from crowding distances measured at two eccentricities at four meridians (Supplementary Figure 1). Observer 1’s *λ* and V4 surface area are more than twice those of Observer 2 while in V1 to V3, the two observers have similar surfaces areas (Figure 2b–c).

Testing the conservation conjecture requires reliable measurement. To assess reliability, we estimated *λ* twice per observer, in separate sessions, and found a high correlation between sessions (*r* = 0.94). Similarly, we measured *A* twice by having two researchers independently draw the retinotopic map boundaries (from the same fMRI data) and found high agreement in surface area between the two researchers (*r* = 0.94, 0.88, 0.74, 0.73 for V1 to V4, respectively). Retinotopic mapping parameters in the early visual cortex are stable across scans^14,15^, which indicates that uncertainty in *A* from scan-to-scan variability is negligible compared to differences in how researchers use heuristics to draw map boundaries. The large-scale features of the retinotopic data, such as the V1/V2 polar angle reversals, are even more stable than individual voxel data, because much of the random measurement noises in individual voxels cancels out in estimating location of large features.

Relating surface area to crowding, we plot each observer’s *A* vs their *λ*, separately for V1 to V4 (Figure 3a). If a map conserves crowding distance *c* across observers, then *λ* will scale with *A*. Only V4 shows high correlation between *λ* and *A* (*r* = 0.65). In V1 to V3, there is no relationship between *λ* and *A*, as indicated by the practically zero correlation coefficients and the circular covariance ellipses in the scatter plots (Figure 3b). Conservation predicts that *A* will explain λ. In fact, V4 area accounts for much (0.43) of the lambda variance, and the areas of V1 to V3 account for none (Figure 3c).

The slope of the conservation line allows us to estimate the required spacing between letters on the V4 map. The slope *k* is 0.54 [CI 0.52 – 0.57] letters per mm^2^ which implies that the cortical crowding distance c is 1.36 [CI 1.33 – 1.39] mm. According to the conservation model, this single number, 1.4 mm, is the required letter-to-letter spacing for all the people we tested, despite very different map sizes and crowding distances. This value is robust to variations in the assumed parameter values used to compute *λ* (Supplementary Analyses 1 and 2).

*Why is cortical crowding distance conserved?* Our main finding is that despite large individual differences in psychophysical crowding distance and map size, crowding distance is conserved on the V4 map but not on V1 to V3. Both crowding and V4 are thought to be associated with mid-level visual processes^16,17^. We speculate that the 1.4 mm crowding distance is conserved because there is a computational unit composed of a fixed number of V4 neurons needed to isolate and recognize an object. A fixed neural count implies a fixed area, which we estimate to be *A*/*λ* = 2 mm^2^ of cortex. This is analogous to a V1 hypercolumn analyzing local scale and orientation, with an area of 1.5 mm^2^ in human^18^. In seeking a link between behavior and brain measures, we emphasized anatomy (map size) rather than physiology (neural response properties). The measured map size is insensitive to task, stimulus, and attentional state (see Extended Figure 9 in ref [^19^]). Previous physiological methods have yielded ambiguous results linking crowding with various visual maps, including V1^20^, V2^21,22^, V3^21^, and V4^23,24^ (Supplementary Table 1). Physiological studies of crowding, and computational models based on physiology, tend to compare neural responses to a target with different flanker conditions, such as flankers present vs absent or near vs far^20,23,24^. A challenge to this approach is that any change of the visual stimulus will have some effect on neural responses in most visual areas. As a result, the particular metric calculated from the neural responses by the experimenter, such as target decodability from a set of sampled neurons^23^ or BOLD response magnitude^20^, is likely to be affected in most visual areas. This challenges the identification of a single brain area as the locus of crowding. This challenge is unsurprising since object recognition relies on computations spanning much of the visual system^25^. In contrast, our search for a neural basis of crowding measured anatomy rather than physiology and exploited individual differences, measuring both crowding and maps in the same 49 individuals. Unlike the physiological approach, our approach showed a sharp divide: no relationship between crowding psychophysics and map size in V1 to V3, and a strong relationship in V4. We interpret this sharp divide as evidence for a spacing bottleneck in recognition: Although image recognition involves many visual areas, the *threshold* spacing — or crowding distance — is determined by characteristics of only the most spacing-sensitive visual area, which our study identifies as V4.

We used individual differences to discover a strong connection between behavior and V4 size. Individual differences can be a useful tool to define visual mechanisms^26^. Individual variations are also important in their own right. Extreme individual variation becomes a disorder, as in dyslexia and dyscalculia, which are associated with unusually large crowding distances^27,28^. Our discovery that the size of the V4 map predicts crowding might help understand these disorders.

## Methods

### Participants

Fifty participants (32 females, 18 males, mean age 27 years) were recruited from New York University. All participants had normal or corrected-to-normal vision. The participants completed one fMRI session to measure retinotopic maps (45 min) and two behavioral sessions to measure crowding distance (30 min each). One participant was dropped from the analysis due to incomplete fMRI data which did not allow for reliable estimation of retinotopic maps. All participants provided written informed consent and approved the public release of anonymized data. The experiment was conducted under the Declaration of Helsinki and was approved by the NYU ethics committee on activities involving human participants.

### Pilot study

We conducted a pilot study with 26 participants and reported the results in a conference abstract^1^. The new experiments reported here were conducted to include test-retest measures, obtain a larger number of participants, and assess replicability. Eight of the pilot subjects were re-tested in the main experiment including both new crowding data and new retinotopic mapping. We performed the same analysis of the pilot data as presented in the main text. The results reported in the pilot study are largely independent and highly consistent with those reported in the main text (Supplementary Figure 3).

### fMRI stimulus display

Participants viewed a retinotopic mapping stimulus in the MRI scanner using a ProPixx DLP LED projector (VPixx Technologies Inc., Saint-Bruno-de-Montarville, QC, Canada). The stimulus image was projected onto an acrylic back-projection screen (60 cm × 36.2 cm) in the scanner bore. The projected image had a resolution of 1920 × 1080 and a refresh rate of 60 Hz. The display luminance was 500 cd/m^2^. Participants viewed the screen at 83.5 cm (from eyes to the screen) using an angled mirror that was mounted on the head coil.

### fMRI Stimuli

The mapping stimulus was generated in MATLAB 2017a and was presented using the Psychophysics Toolbox v3^2^ and custom vistadisp software (https://github.com/WinawerLab/vistadisp) on an iMac computer. For 27 participants, stimulus images were shown within a sweeping bar aperture, identical to the stimulus in Himmelberg et al^3^. For 33 participants, the stimulus image patterns were shown within two types of apertures in alternative scans, a sweeping bar and a wedge+ring. These stimulus apertures were similar to the ones used in the NSD dataset^4^. Unlike NSD, our stimuli changed location in discrete steps and had a larger maximum eccentricity. In short, there were six runs of retinotopic mapping, each lasting 5 minutes (300 TRs of 1 sec). In recent work we found that the inclusion of the wedge+ring aperture was important for getting reliable estimates of pRFs centered near the fovea^3^. Each aperture type was shown three times in an interleaved order. The carrier image pattern was presented with a 3 Hz temporal frequency and was composed of colorful objects, faces, and scenes at multiple scales. These images were superimposed on an achromatic pink-noise (1/f) background^5^. The stimulus image pattern was windowed within a circular aperture (12.4° of maximum radius). The apertures were superimposed on a polar fixation grid placed upon a uniform gray background, with a red or green dot at the center (3 pixels, or 0.07°). Participants completed a fixation task to ensure that they were maintaining central fixation and remained alert throughout the scan. Participants were required to respond, via button press, when the central fixation dot changed from green to red, or vice versa.

### Data acquisition and preprocessing

Structural and functional data were acquired on a 3T Siemens MAGNETOM Prisma MRI scanner (Siemens Medical Solutions, Erlangen, Germany) at the Center for Brain Imaging at NYU. Both the structural and functional images were acquired using a Siemens 64-channel head coil. MPRAGE anatomical images were acquired for each participant (TR, 2400 ms; TE, 2.4 ms; voxel size, 0.8mm^3^ isotropic; flip angle, 8°), and were auto-aligned to a template to ensure a similar slice prescription for all participants. Additionally, two distortion maps were acquired to correct susceptibility distortions in the functional images: one spin-echo image with anterior-posterior (AP) and one with posterior-anterior (PA) phase encoding. Anatomical and functional preprocessing was performed using fMRIPrep v.23.1.2^6^. For each participant and each run, fMRIprep produced a BOLD time series on the participant’s native freesurfer surface.

### Population receptive field model

We first averaged the time-series data from the three repetitions of bar aperture and three repetitions of wedge and ring apertures resulting in two functional time series. For the 27 participants who saw images only through a bar aperture, we averaged all 6 runs. We used these average data to fit the pRF model. The analysis was conducted using vistasoft (https://github.com/WinawerLab/prfVista). A pRF is modeled as a circular 2D-Gaussian, as described in Dumoulin and Wandell^7^. The Gaussian is parameterized by values at each vertex for x, y, and σ. The x and y parameters specify the center position of the 2D-Gaussian in the visual field. The σ parameter, the standard deviation of the 2D-Gaussian, specifies the size of the receptive field. The 2D-Gaussian is multiplied pointwise by the stimulus contrast aperture (apertures were prepared at a resolution of 101 pixels × 101 pixels), and then convolved with a hemodynamic response function (HRF) to predict the BOLD percent signal change. The HRF is parameterized by 5 values, describing a difference of two gamma functions, as used previously^7^. The HRF was assumed to be the same across vertices within a participant but differed among participants. We use a two-stage coarse-to-fine approach described in detail by Dumoulin and Wandell^7^, and the addition of the HRF fit is described in detail by Harvey and Dumoulin^8^

### Defining the size of visual areas (V1-V4)

Two authors, JWK and BSQ, independently defined ROIs (V1-V4) by hand using neuropythy (https://github.com/noahbenson/neuropythy)^9^. To define visual maps, researchers followed the heuristics of Wandell^10^ for V1-V3, and of Winawer and Witthoft^11^ for V4. To match the maximum eccentricity of the psychophysical stimuli we restricted all maps to a maximum eccentricity of 10 deg. The size of each map was calculated as the sum of the surface areas of all vertices included in the map on the mid-gray cortical meshes, defined by FreeSurfer as the surfaces hallway between the meshes at the gray/white interface and the pial/gray interface. Finally, each map surface area was summed across hemispheres.

### Measuring crowding distance

Data were acquired with the CriticalSpacing software^12^, allowing for reliable and relatively fast measurement of crowding distances. Our crowding database consists of measurements of crowding distance with the Sloan font with radial flankers. We measured crowding at 8 different peripheral locations in the visual field: 2 eccentricities (5, 10 deg) along the four cardinal meridians (upper, lower, left and right). Each observer participated in two crowding sessions (each lasting about 40 min). Sessions were separated at least by a day with a maximum of five days. In session one first block showed stimuli at 5 deg and the second showed stimuli at 10 deg. In the second session the order was reversed.

### Crowding stimulus display

Each testing session was completed on an Apple iMac 27” with an external monitor for stimulus presentation. Screen resolution was 5120 × 2880. Apple iMac has AMD graphics for optimal compatibility with the Psychtoolbox imaging software. The Systems Preference: Displays: Brightness slider was set (by calling MacDisplaySettings.m in the Psychtoolbox) to 0.86 (range 0 to 1) to achieve a white background luminance of about 250 cd/m^2^. The observer viewed the screen binocularly at 40 cm distance from the screen. Stimuli were rendered in MATLAB 2021 using the Psychtoolbox^2^

### Crowding stimuli

To measure crowding we show a three-letter trigram. For each trial, the three letters are drawn randomly, without replacement, from the 9 possible letters. Letters are rendered as black in the Sloan font, presented on a uniform white background of about 250 cd/m^2^. For the Sloan font we use the letters “DHKNORSVZ” as possible targets and flankers. For crowding, each trigram is always arranged radially. Each testing session is about 30 minutes, measuring crowding for targets at two eccentricities, 5° and 10°. The eccentricities are measured in separate blocks of about 15 min each. Each block measures crowding thresholds in the four cardinal directions, with four interleaved staircases. Fixation is displayed until the observer presses a key to initiate the trial. Then the letter trigram appears on the screen for 150 ms and the computer waits for the observer to identify the middle letter by choosing the letter using a mouse click. Letter choices appear on the screen after stimulus offset. Observers are instructed to return their eyes to fixation before clicking their response. The computer waits indefinitely for each response. 300 ms after the observer’s response, the next trial begins as soon as the observer fixates the center of the crosshair for 250 ms.

### Eye tracking

For all thresholds, we controlled for eye movements. To measure fixation, we used an Eyelink 1000 eye tracker (SR Research, Ottawa, Ontario, Canada) with a sampling rate of 1000 Hz. To allow for short viewing distance (40 cm) we used their Tower mount setup with a 25 mm lens mounted on the Eyelink camera. We allowed participants to move their eyes within a 1.5-degree radius from fixation. We checked eye position during the stimulus presentation. If during the stimulus presentation, observers moved their eyes more than 1.5 deg away from the fixation, the fixation cross turned red, and observers were asked to repeat the trial. Each threshold was estimated based on 35 trials with correct fixation. On average, 10% of trials had to be repeated.

### Measuring crowding distance

Pelli’s^12^ procedure estimates the crowding distance. Letter spacing is controlled by QUEST. Spacing scales with letter size, maintaining a fixed ratio of 1.4:1. We set the guessing rate parameter gamma to the reciprocal of the characters in the test alphabet for that font, usually 9. We set the “finger error” probability delta to 1%, so that QUEST can better cope with an occasional reporting mistake. At the end of each block, QUEST estimates the threshold (crowding distance). We devote 35 trials to measure the threshold for a condition. In each block we randomly interleaved the four meridian locations, to get a threshold for each condition, while keeping the observer uncertain as to which condition is present on each trial.

### Calculating the Bouma factor *b*

For each participant we measured 16 crowding distances (2 eccentricities × 4 cardinal meridians × 2 sessions). For each session, crowding distances were converted to Bouma factors by dividing them by the target eccentricity. (This calculation omits *φ*_0_ because it’s negligible, 0.24 deg << 2.5 deg.) Next, we estimated one Bouma factor for each session by taking the geometric mean across all 8 visual field locations. The final estimate for each observer was calculated as the geometric mean of Bouma factors from sessions 1 and 2.

### Calculating the number of uncrowded letters *λ*

We used the Bouma law to calculate the number *λ* of letters that fit into the visual field without crowding. The Bouma law states that the radial crowding distance *s*_r_, in deg, grows linearly with eccentricity *φ*, in deg,

(4) $$s_{r}=(\varphi_{0}+\varphi )b,$$

where *φ*_0_ and *b* are fitted constants. Tangential crowding distance *s*_t_ is generally found to be proportional to radial crowding distance,

(5) $$s_{t}=\frac{s_{r}}{\alpha }$$

where *α* (typically 2) is the ratio of radial-to-tangential crowding distances^13^.

When letters are packed as tightly as possible without crowding, the local density, in letters per deg^2^, is (*s*_r_*s*_t_)^−1^, the reciprocal of the product of the radial and tangential crowding distances. The number *λ* of uncrowded letters in the visual field is the integral of that density over the visual field,

(6) $$\lambda =\int_{\varphi =0\text{deg}}^{\varphi_{\text{max}}}\int_{\theta =0}^{2\pi }\frac{\varphi }{s_{r}s_{t}}d\theta d\varphi ,$$

where *φ* is the eccentricity (in deg), *φ*_max_ is the maximum extent of the visual field (in deg), *θ* is the polar angle, and *s*_r_ and *s*_t_ are the radial and tangential crowding distances (in deg). Integrating yields a formula,

(7) $$\lambda =2\pi \alpha b^{-2}\left(In\left(1+\frac{\varphi_{\text{max}}}{\varphi_{0}}\right)-\frac{1}{1+\frac{\varphi_{0}}{\varphi_{\text{max}}}}\right).$$

The last term is an approximately logarithmic function of the ratio *φ*_max_/*φ*_0_ (Supplementary Analysis 2). We set *φ*_max_ = 10 deg, corresponding to the maximum eccentricity of both the psychophysical and retinotopic data. We took measured values, *φ*_0_ = 0.24 deg and *α* = 2, from our prior study^14^. With these values, the formula reduces to

(8) $$\lambda =\frac{34.9}{b^{2}}.$$

Our measure of interest, *c*, the cortical letter spacing at threshold separation, depends on *λ*. We analyzed the sensitivity of *c* to the values of *α* and *φ*_0_ (Supplementary Analysis 1).

### Relationship between *A and λ*

**Figure 3.**
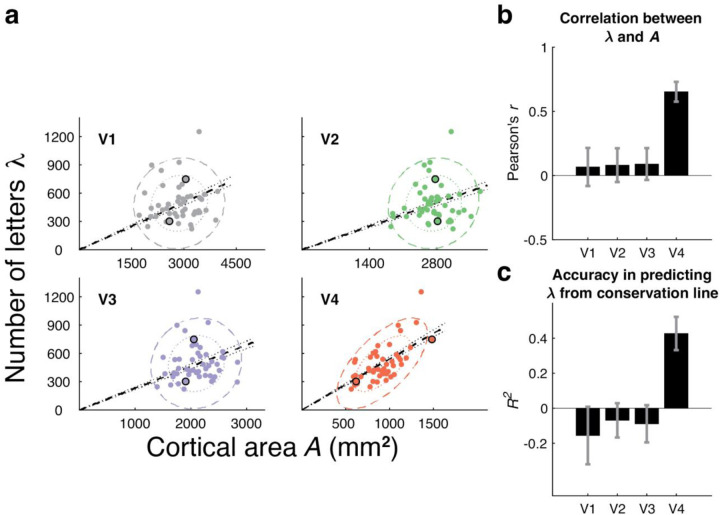
A test of cortical crowding distance conservation in V1 to V4. **a,** Each panel plots one point per observer as *λ*, the number of letters that fit into the uncrowded visual field (up to 10 deg eccentricity) vs *A*, the surface area of a visual map (summed across hemispheres), for 49 observers. Black outlines around two dots in each panel show data for the two observers from Figure 2. The colored ellipses are the 1-SD and 2-SD contours from the covariance ellipses. The black dashed lines are the fits by regression with one parameter, slope, i.e., the prediction assuming conservation. The dotted black line shows 68th percentile of the conservation fit. The slope of this line has units of letters/mm^2^ and is equal to 1/c^2^, where c is the cortical crowding distance. **b,** The correlation between *λ* and *A* for V1-V4. **c,** The *R*^2^ value is the coefficient of determination, the amount of variance in *λ* explained by assuming conservation (one-parameter fit). To estimate confidence intervals in Panels A (conservation line), B, and C, we bootstrapped across observers (n = 10,000).

We quantified the relationship between *A*, cortical map surface area, and *λ*, number of letters in the uncrowded visual field, in two ways. First, we computed the correlation (Pearson’s *r*) and covariance between the two variables. These calculations treat the two variables symmetrically. We visualize the covariance ellipses at 1-SD and 2-SD, scaling the x and y axes so that one standard deviation of each variable is the same length (Figure 3a). This ensures that the covariance ellipses are circular when the correlation is 0. Second, to explicitly test for conservation, we fit a linear model to *A* (independent variable) and *λ* (dependent variable), with the intercept set to 0, and asked how much of the variance in *λ* is explained (Figure 3c). Note that the variance explained can be negative, because a line through the origin may be a worse predictor (larger residuals in *λ*) than just assuming the mean (a horizontal line). For both the correlation coefficient and the variance explained, 68% confidence intervals were calculated by bootstrapping participants with replacement (*n* = 10000).

## Supplementary Material

1

## Figures and Tables

**Figure 1.**
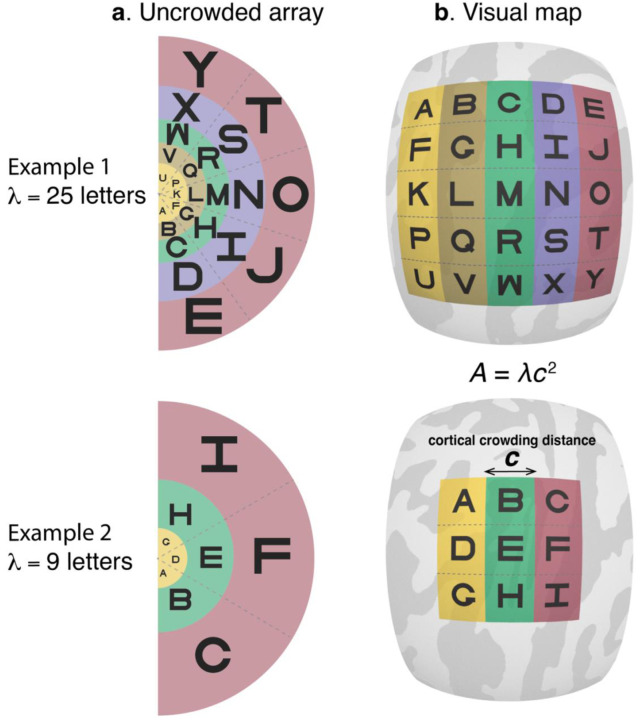
Conservation of cortical crowding distance. **a,** The left column shows two hypothetical examples of uncrowded visual fields. In each case, the letters are spaced at the minimum distance necessary for successful recognition. The lower spacing in the upper row indicates that this hypothetical observer has a small crowding distance, and hence can fit more letters into the uncrowded visual field (*λ* = 25 vs *λ* = 9). **b,** The right column shows the representation of these two visual fields in retinotopic maps on the cortical surface, assuming cortical conservation of crowding. According to this conjecture, the greater *λ* for the first observer is explained by a larger cortical map. The center-to-center letter spacing on the cortex in mm is *c,* the same for both observers. The surface area *A* of the map is *λc*^2^, the product of the number of letters and the area per letter. Hence the conservation conjecture predicts that the number of letters in the uncrowded field is proportional to surface area.

**Figure 2.**
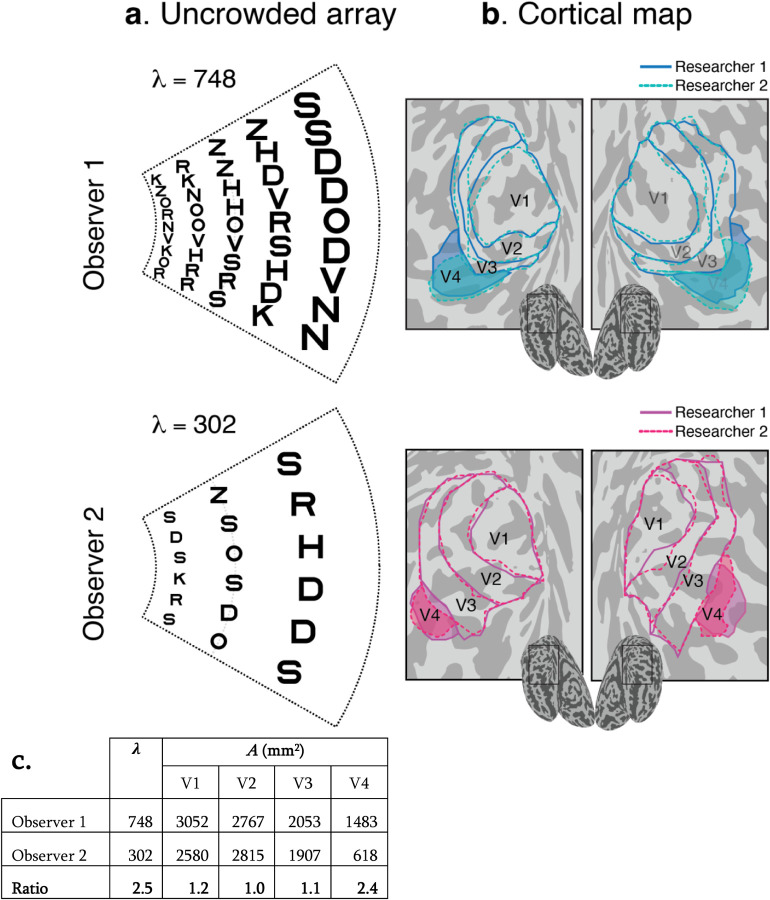
Individual differences in *λ* and map size *A*. **a,** For each observer, we show a portion of the visual field with letters spaced according to their Bouma factor. Observer 1 has a small Bouma factor (0.21 radially) and can individuate *λ* = 748 letters. Observer 2 has a large Bouma factor (0.34 radially) and individuates less than half as many letters, *λ* = 302. The portion of the visual field shown is 2.5° to 10° eccentricity, ±25° polar angle. The *λ* values count letters only within 10° eccentricity, the extent of our psychophysics and retinotopy. **b,** Retinotopic map boundaries (V1 to V4) from both hemispheres of the same two observers. Visual maps were independently drawn by two researchers (dashed and solid lines). The numbers in the table indicate the surface area *A*, in mm^2^ of the bilateral retinotopic maps, averaged across the two researchers. **c.** Observer 1’s *λ* is more than twice as big as Observer 2’s. Observer 1’s V4 is more than twice as big as Observer 2’s (Supplementary Figure 2) while V1 to V3 sizes are similar.

## References

[1] Song S., Levi D. M. & Pelli D. G. A double dissociation of the acuity and crowding limits to letter identification, and the promise of improved visual screening. Journal of Vision 14, 3 (2014). 10.1167/14.5.3 PMC4021854 24799622

[2] Geisler W. S. Physical limits of acuity and hyperacuity. J Opt Soc Am A 1, 775–782 (1984). 10.1364/josaa.1.000775 6747742

[3] Pelli D. G. Crowding and eccentricity determine reading rate. Journal of Vision 7, 20 (2007). 10.1167/7.2.20 18217835

[4] Rosenholtz R., Huang J., Raj A., Balas B. J. & Ilie L. A summary statistic representation in peripheral vision explains visual search. Journal of Vision 12 (2012). 10.1167/12.4.14 PMC4032502 22523401

[5] Kwon M., Bao P., Millin R. & Tjan B. S. Radial-tangential anisotropy of crowding in the early visual areas. Journal of Neurophysiology 112, 2413–2422 (2014). 10.1152/jn.00476.2014 25122703 PMC4233277

[6] Kurzawski J. W. The Bouma law accounts for crowding in 50 observers. Journal of Vision 23, 6 (2023). 10.1167/jov.23.8.6 PMC10408772 37540179

[7] Kurzawski J. W. EasyEyes — A new method for accurate fixation in online vision testing. Front. Hum. Neurosci. 17 (2023). 10.3389/fnhum.2023.1255465 PMC10718086 38094145

[8] Toet A. & Levi D. M. The two-dimensional shape of spatial interaction zones in the parafovea. Vision Res 32, 1349–1357 (1992). 10.1016/0042-6989(92)90227-a 1455707

[9] Veríssimo I. S., Hölsken S. & Olivers C. N. L. Individual differences in crowding predict visual search performance. Journal of Vision 21, 29 (2021). 10.1167/jov.21.5.29 34038508 PMC8164367

[10] Anstis S. M. Letter: A chart demonstrating variations in acuity with retinal position. Vision Res 14, 589–592 (1974). 10.1016/0042-6989(74)90049-2 4419807

[11] Pelli D. G. Crowding: a cortical constraint on object recognition. Curr Opin Neurobiol 18, 445–451 (2008). 10.1016/j.conb.2008.09.008 18835355 PMC3624758

[12] Strasburger H. On the cortical mapping function - Visual space, cortical space, and crowding. Vision Res 194, 107972 (2022). 10.1016/j.visres.2021.107972 35182892

[13] Benson N. C. Variability of the Surface Area of the V1, V2, and V3 Maps in a Large Sample of Human Observers. J. Neurosci. 42, 8629–8646 (2022). 10.1523/JNEUROSCI.0690-21.2022 36180226 PMC9671582

[14] Himmelberg M. M. Cross-dataset reproducibility of human retinotopic maps. NeuroImage 244, 118609 (2021). 10.1016/j.neuroimage.2021.118609 34582948 PMC8560578

[15] van Dijk J. A., de Haas B., Moutsiana C. & Schwarzkopf D. S. Intersession reliability of population receptive field estimates. NeuroImage 143, 293–303 (2016). 10.1016/j.neuroimage.2016.09.013 27620984 PMC5139984

[16] Pelli D. G. & Tillman K. A. The uncrowded window of object recognition. Nat Neurosci 11, 1129–1135 (2008). 10.1038/nn.2187 18828191 PMC2772078

[17] Roe A. W. Toward a unified theory of visual area V4. Neuron 74, 12–29 (2012). 10.1016/j.neuron.2012.03.011 22500626 PMC4912377

[18] Garcia-Marin V., Kelly J. G. & Hawken M. J. Neuronal composition of processing modules in human V1: laminar density for neuronal and non-neuronal populations and a comparison with macaque. Cereb Cortex 34, bhad512 (2024). 10.1093/cercor/bhad512 38183210 PMC10839852

[19] Allen E. J. A massive 7T fMRI dataset to bridge cognitive neuroscience and artificial intelligence. Nat Neurosci 25, 116–126 (2022). 10.1038/s41593-021-00962-x 34916659

[20] Millin R., Arman A. C., Chung S. T. L. & Tjan B. S. Visual Crowding in V1. Cereb Cortex 24, 3107–3115 (2014). 10.1093/cercor/bht159 23833128 PMC4224237

[21] Bi T., Cai P., Zhou T. & Fang F. The effect of crowding on orientation-selective adaptation in human early visual cortex. Journal of Vision 9, 13.11–10 (2009). 10.1167/9.11.13 20053076

[22] Freeman J. & Simoncelli E. P. Metamers of the ventral stream. Nat Neurosci 14, 1195–1201 (2011). 10.1038/nn.2889 21841776 PMC3164938

[23] Henry C. A. & Kohn A. Feature representation under crowding in macaque V1 and V4 neuronal populations. Curr Biol 32, 5126–5137.e5123 (2022). 10.1016/j.cub.2022.10.049 36379216 PMC9729449

[24] Motter B. C. Stimulus conflation and tuning selectivity in V4 neurons: a model of visual crowding. Journal of Vision 18, 15 (2018). 10.1167/18.1.15 PMC5783327 29362808

[25] DiCarlo J. J., Zoccolan D. & Rust N. C. How does the brain solve visual object recognition? Neuron 73, 415–434 (2012). 10.1016/j.neuron.2012.01.010 22325196 PMC3306444

[26] Wilmer J. B. How to use individual differences to isolate functional organization, biology, and utility of visual functions; with illustrative proposals for stereopsis. Spat Vis 21, 561–579 (2008). 10.1163/156856808786451408 19017483 PMC2586597

[27] Bertoni S., Franceschini S., Ronconi L., Gori S. & Facoetti A. Is excessive visual crowding causally linked to developmental dyslexia? Neuropsychologia 130, 107–117 (2019). 10.1016/j.neuropsychologia.2019.04.018 31077708

[28] Castaldi E., Turi M., Gassama S., Piazza M. & Eger E. Excessive visual crowding effects in developmental dyscalculia. Journal of Vision 20, 7 (2020). 10.1167/jov.20.8.7 PMC7438630 32756882

