# Supplementary material for: Human V4 size predicts crowding distance
Source: bioRxiv. 2025 Feb 28:2024.04.03.587977. Originally published 2024 Apr 5. Preprint. [Version 4] doi: 10.1101/2024.04.03.587977 (PMC11014589; doi:10.1101/2024.04.03.587977)
Supplement: 1 [file NIHPP2024.04.03.587977v4-supplement-1.pdf]

bioRxiv preprint doi: https://doi.org/10.1101/2024.04.03.587977; this version posted February 28, 2025. The copyright holder for this preprint (which was not certified by peer review) is the author/funder, who has granted bioRxiv a license to display the preprint in perpetuity. It is made available under aCC-BY 4.0 International license.

Supplementary materials

Supplementary Figure 1. Retinotopic maps for Observers 1 and 2

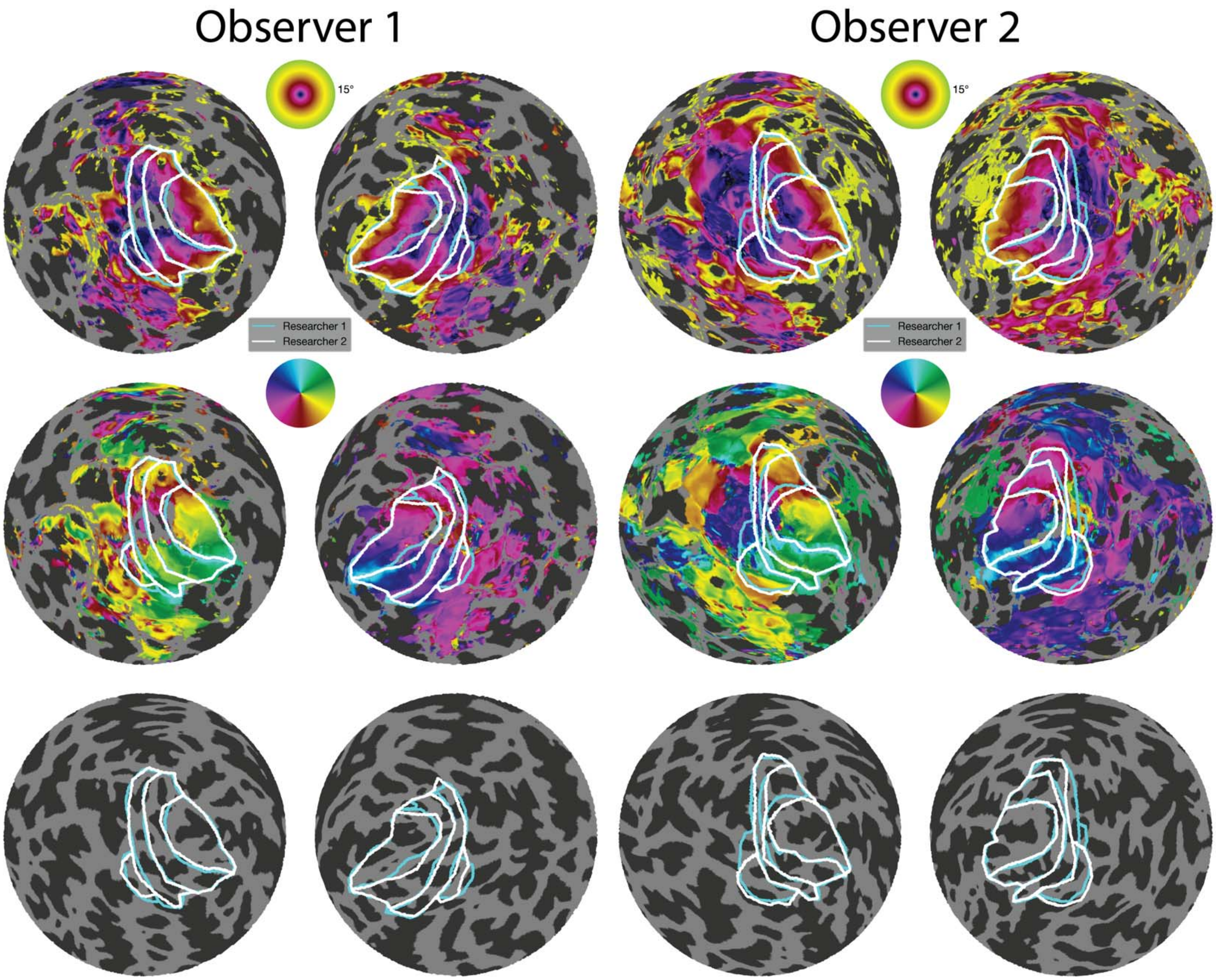

Supplementary Figure 1. Retinotopic maps for Observers 1 and 2. For each observer we show flattened representations of their left and right hemispheres. The overlays show eccentricity (top), polar angle (middle) and underlying thresholded cortical curvature (bottom; dark regions are sulci, light regions are gyri). The two sets of outlines (blue and white) are the map boundaries drawn independently by two researchers. In rows 1 and 2, the color overlays are thresholded by pRF variance explained > 10%. Maps for all participants are available as images in Supplementary Data 1 (retinotopicMaps.zip).

bioRxiv preprint doi: https://doi.org/10.1101/2024.04.03.587977; this version posted February 28, 2025. The copyright holder for this preprint (which was not certified by peer review) is the author/funder, who has granted bioRxiv a license to display the preprint in perpetuity. It is made available under aCC-BY 4.0 International license.

Supplementary Figure 2. Model fits of vs *A* for V1 to V4, based on map boundaries from single researchers.

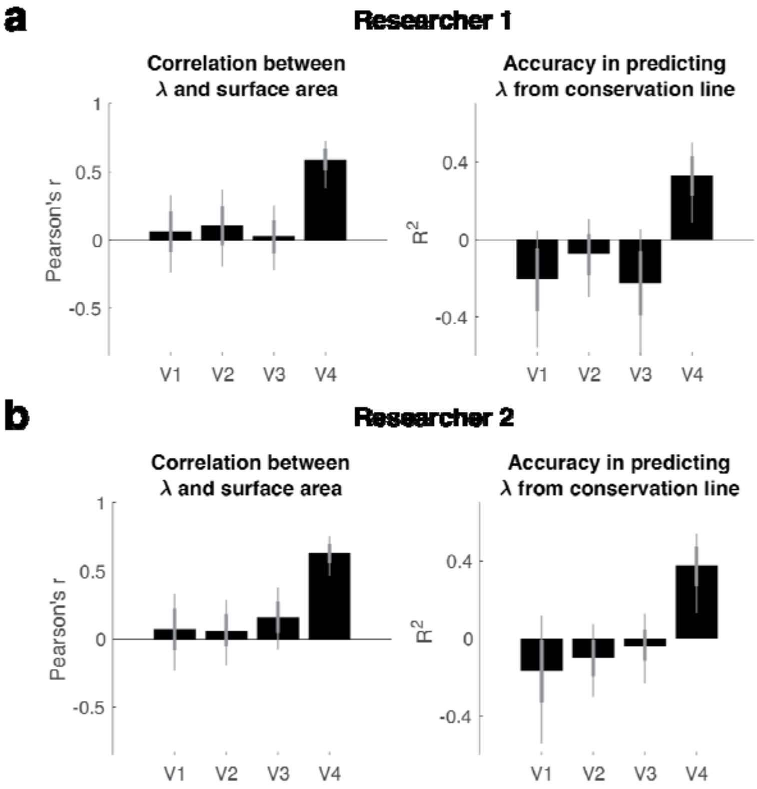

Supplementary Figure 2. Model fits of vs *A* for V1 to V4, based on map boundaries from single researchers. Panel a shows the correlation coefficient (left) and variance explained from linear regression with 0 intercept (right) when using cortical map definitions from Researcher 1. Panel b is the same but for Researcher 2. The thick and thin error bars are 68% and 95% confidence intervals. The results can be compared to Figure 5b and 5c in the main text, which compute the same statistics for map surface area averaged across the two researchers.

bioRxiv preprint doi: https://doi.org/10.1101/2024.04.03.587977; this version posted February 28, 2025. The copyright holder for this preprint (which was not certified by peer review) is the author/funder, who has granted bioRxiv a license to display the preprint in perpetuity. It is made available under aCC-BY 4.0 International license.

Supplementary Figure 3.

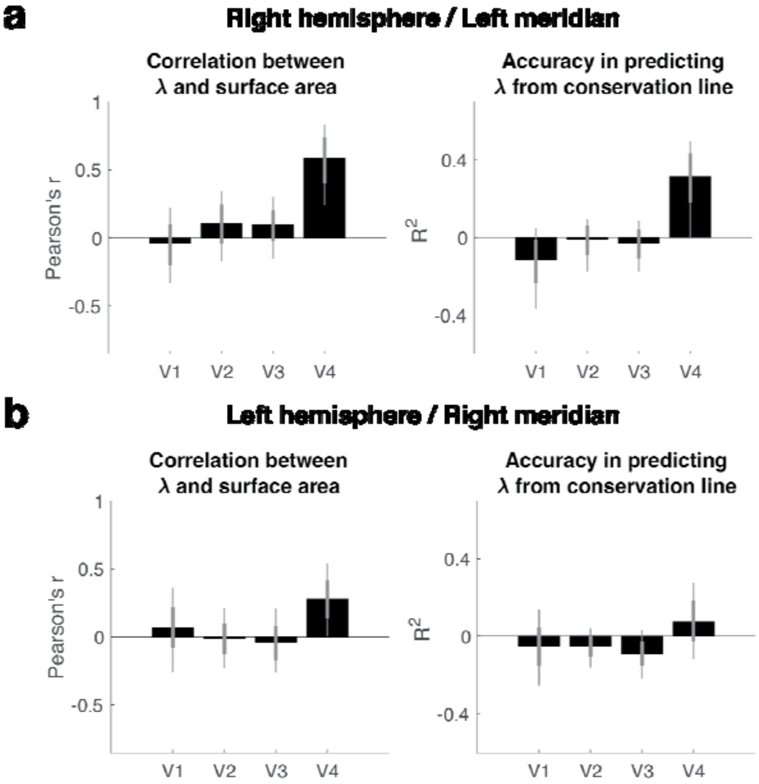

Supplementary Figure 3. Model fits of vs *A* for V1 to V4, separated by cortical hemisphere. Both panels are as in Supplementary Figure 2, except that: (1) the map surface area is computed only for the right hemisphere (panel a) or left hemisphere (panel b) and is derived from crowding data only on the left horizontal meridian (Panel a) or right horizontal meridian (panel b). Surface area measures are averaged across the two researchers.

bioRxiv preprint doi: https://doi.org/10.1101/2024.04.03.587977; this version posted February 28, 2025. The copyright holder for this preprint (which was not certified by peer review) is the author/funder, who has granted bioRxiv a license to display the preprint in perpetuity. It is made available under aCC-BY 4.0 International license.

Supplementary Figure 4.

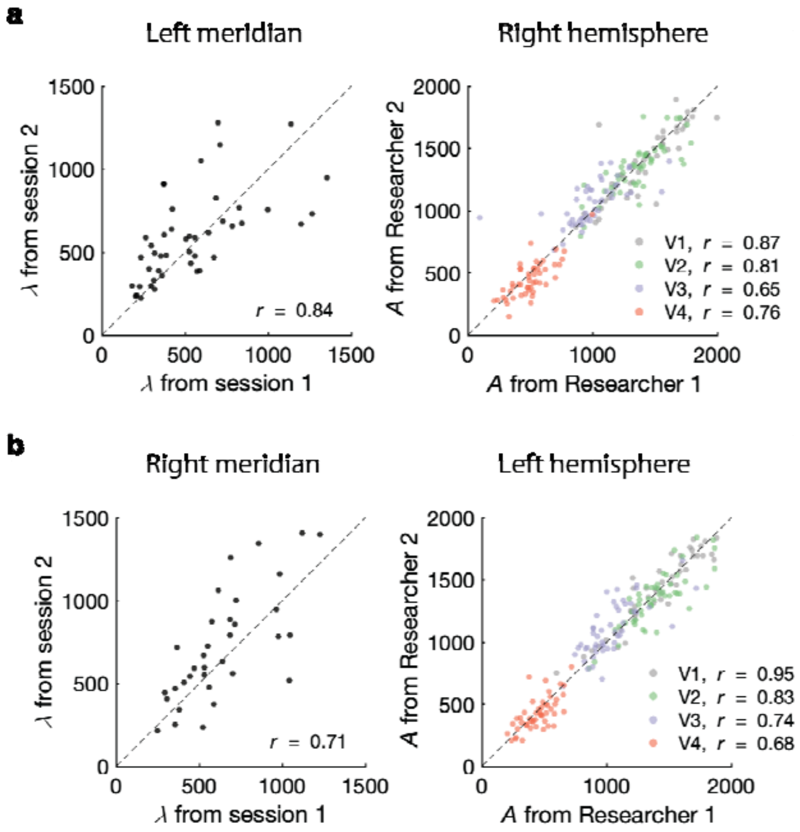

Supplementary Figure 4. Measurement reliability of crowding distance and map size separated by hemisphere and visual field location. Both panels are the same as in Figure 4, except that panel a plots from the left meridian and right hemisphere, while panel b plots the data from right meridian and left hemisphere.

bioRxiv preprint doi: https://doi.org/10.1101/2024.04.03.587977; this version posted February 28, 2025. The copyright holder for this preprint (which was not certified by peer review) is the author/funder, who has granted bioRxiv a license to display the preprint in perpetuity. It is made available under aCC-BY 4.0 International license.

Supplementary Figure 5.

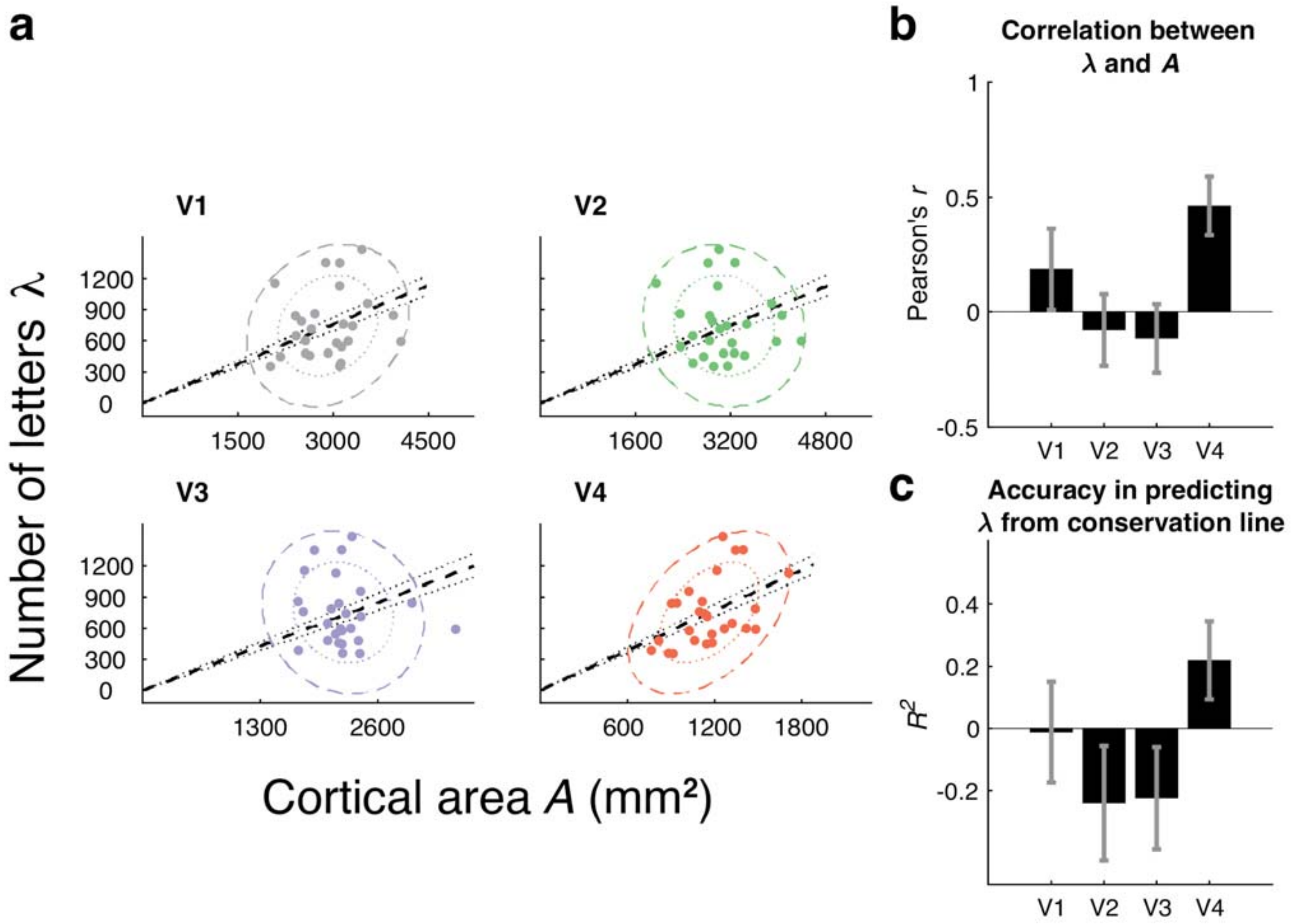

d

		Pilot	Main
Methods	Number of participants	26	49 (50 planned)
	Nature of research	Exploratory	Confirmatory
	Crowding sessions per subject	1	2
	Number of researchers defining map boundaries	1	2
	pRF stimulus design for better mapping of the fovea[1]	no	yes
	Gaze contingent stimulus display	no	yes
	Removing trials with fixation errors	no	yes
Results	k (letters / mm^2) in V4	0.64 [CI 0.60 - 0.69]	0.54 [CI 0.52 - 0.57]
	c (mm per letter) in V4	1.25 [CI 1.20 - 1.30]	1.36 [CI 1.33 - 1.39]
	r (crowding distance, surface area) V4	0.46	0.66
	R^2 (conservation prediction), V4	0.22 [CI 0.09 - 0.33]	0.43 [CI 0.33 - 0.50]

Supplementary Figure 5. A comparison of pilot and main experiments a, Number of letters λ vs cortical surface area A for the 26 observers in the pilot dataset. Otherwise plotted as in Figure 3a. b, The correlation between λ and *A* for V1 to V4 in the pilot dataset. Otherwise plotted as in **Figure 3bc**, Variance explained by conservation prediction. Plotted as in **Figure 3c**. d, A comparison of methods and results between the pilot and main experiments.

bioRxiv preprint doi: https://doi.org/10.1101/2024.04.03.587977; this version posted February 28, 2025. The copyright holder for this preprint (which was not certified by peer review) is the author/funder, who has granted bioRxiv a license to display the preprint in perpetuity. It is made available under aCC-BY 4.0 International license.

Supplementary Figure 6.

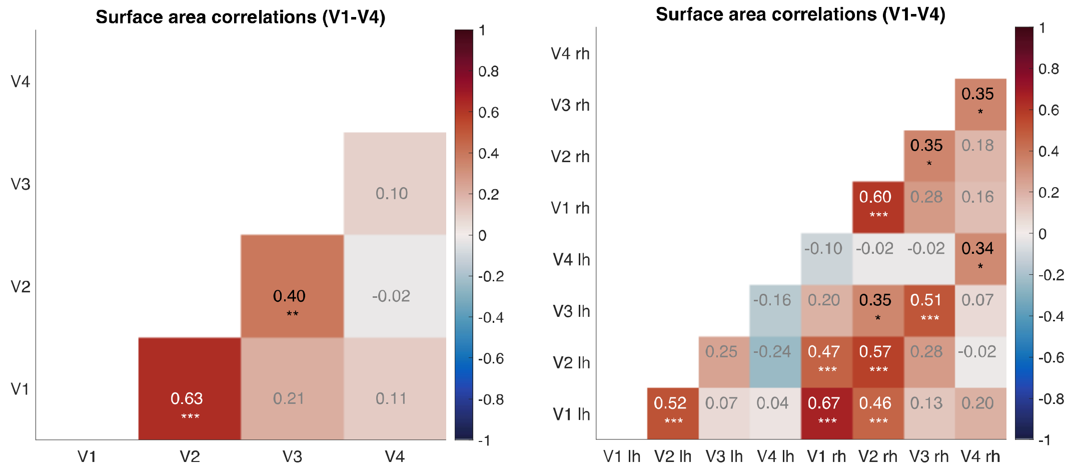

Supplementary Figure 6. Correlations between surface areas of different maps. The left panel shows correlations for bilateral maps. Note that V4 has little correlation with the surface area of V1 to V3. The panel on the right shows the same but separated by hemisphere. The values in the cells are Pearson correlations (n=49). The asterisks indicate statistical significance of the null hypothesis test that the correlation between surface areas of two maps is exactly 0. One asterisk is $p < 0.05$, two asterisks is $p < 0.01$, and three asterisks mean $p < 0.001$.

bioRxiv preprint doi: https://doi.org/10.1101/2024.04.03.587977; this version posted February 28, 2025. The copyright holder for this preprint (which was not certified by peer review) is the author/funder, who has granted bioRxiv a license to display the preprint in perpetuity. It is made available under aCC-BY 4.0 International license.

Supplementary Analysis 1: Sensitivity analysis of the estimated cortical crowding distance *c*

Effects of α and φ_0 on c.

We concluded that cortical crowding distance is conserved on the V4 map, meaning that there is a single value for *c* (cortical crowding distance) across participants, estimated to be 1.4 mm. We estimated this value by regressing λ, number of uncrowded letters, on *A*, the surface area of the V4 map, assuming an intercept of 0 (eq 1 in main text), and then solving for *c* (equation 3 in main text).

Further, we reported uncertainty in *c* from bootstrapping across participants (**Figure 3**). Here we assess how c is affected by variation of λ across participants. The main-text calculation of λ assumed fixed values from our previous work[2] for two parameters: the tangential-to-radial ratio α of crowding distances and the Bouma law intercept φ_0. Here we calculate how the known parameter variations would affect the *c* estimate. We computed *c* 20,000 times, each time assuming different α values with φ_0 fixed (10,000 calculations), or different φ_0 values with α fixed (10,000 calculations). The values for the two parameters were based on the measured distributions, rather than assuming the mean as in the main text. From our prior study, the mean value of α was 2.10 (sd = 0.38), and the mean value of φ_0 was 0.24 (sd 0.05). Supplementary Table 1 below reports the *c* median and 95% confidence intervals from the calculations.

Median [95% CI] estimate of *c*, assuming:<br>α (□ = 2.10, □ = 0.38),<br>φ_0 = 0.24	Median [95% CI] estimate of c, assuming:<br>α = 2.10,<br>φ_0 (□ = 0.24, □ = 0.05)
1.32 [1.14,1.64]	1.36 [1.25,1.45]

Supplementary Table 1. Sensitivity of cortical crowding distance to variation in α and φ_0.

bioRxiv preprint doi: https://doi.org/10.1101/2024.04.03.587977; this version posted February 28, 2025. The copyright holder for this preprint (which was not certified by peer review) is the author/funder, who has granted bioRxiv a license to display the preprint in perpetuity. It is made available under aCC-BY 4.0 International license.

Supplementary Analysis 2. *Effect of* max *on* .

The formula (Eq. 7) for number of uncrowded letters grows approximately logarithmically with max/ 0 (**Supplementary Figure 7,** below). Our decision to measure (in psychophysics and fMRI) out to 10 deg eccentricity was somewhat arbitrary, but we can use equation 7 to consider how many more uncrowded letters might fit in the far periphery. As expected of logarithmic dependence, large changes in the maximum eccentricity produce only modest changes in the number of letters. For instance, doubling the maximum eccentricity max from 10 to 20 deg would increase the number of uncrowded letters by only 13%, and increasing it tenfold, from 10 to 100 deg, would increase by 47%.

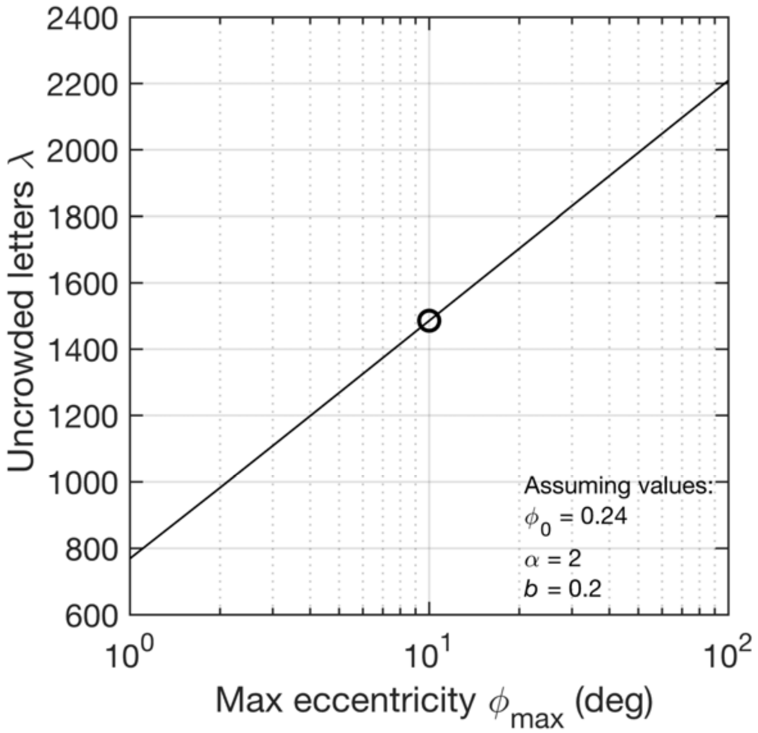

Supplementary Figure 7. Sensitivity of number of uncrowded letters to maximum eccentricity max. Plots the formula (Eq. 7) for vs. max. This paper mostly sets max to 10 deg, shown by the circle. The nearly straight curve shows that the formula's dependence on max is practically logarithmic.

bioRxiv preprint doi: https://doi.org/10.1101/2024.04.03.587977; this version posted February 28, 2025. The copyright holder for this preprint (which was not certified by peer review) is the author/funder, who has granted bioRxiv a license to display the preprint in perpetuity. It is made available under aCC-BY 4.0 International license.

Supplementary Data 1

Please see attached zip file which includes images of visual maps in all 49 subjects.

Supplementary Data 2

Please see attached csv file which includes a table of visual map sizes and crowding measures for all subjects.

bioRxiv preprint doi: https://doi.org/10.1101/2024.04.03.587977; this version posted February 28, 2025. The copyright holder for this preprint (which was not certified by peer review) is the author/funder, who has granted bioRxiv a license to display the preprint in perpetuity. It is made available under aCC-BY 4.0 International license.

Supplementary References

1 Himmelberg, M. M. *et al.* Cross-dataset reproducibility of human retinotopic maps. *NeuroImage* **244**, 118609 (2021). https://doi.org/10.1016/j.neuroimage.2021.118609

2 Kurzawski, J. W. *et al.* The Bouma law accounts for crowding in 50 observers. *Journal of Vision* **23**, 6 (2023). https://doi.org/10.1167/jov.23.8.6
