# Supplementary material for: Conservation of cortical crowding distance across individuals in human V4
Source: bioRxiv. 2024 Apr 29:2024.04.03.587977. Originally published 2024 Apr 5. Preprint. [Version 2] doi: 10.1101/2024.04.03.587977 (PMC11014589; doi:10.1101/2024.04.03.587977)
Supplement: Supplementary file 1 [file NIHPP2024.04.03.587977V2-supplement-1.pdf]

bioRxiv preprint doi: https://doi.org/10.1101/2024.04.03.587977; this version posted April 29, 2024. The copyright holder for this preprint (which was not certified by peer review) is the author/funder, who has granted bioRxiv a license to display the preprint in perpetuity. It is made available under aCC-BY 4.0 International license.

Supplementary materials

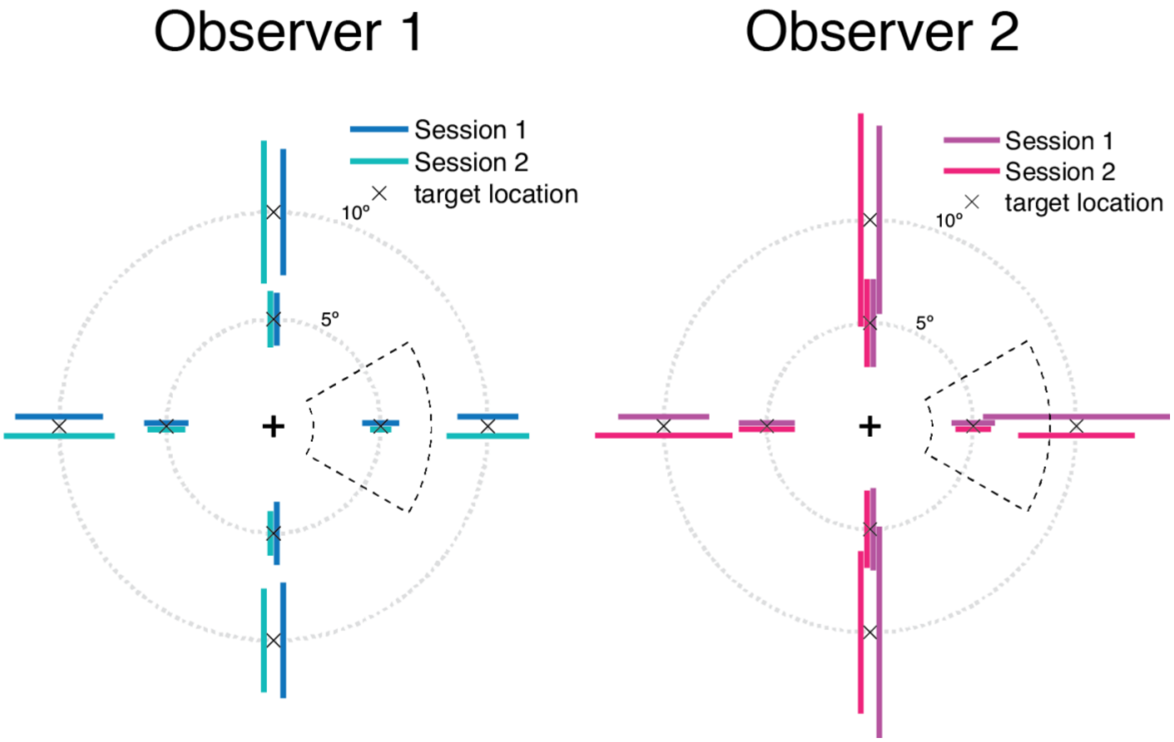

Supplementary Figure 1. Crowding distances for Observers 1 and 2. The crowding distance was measured radially at two eccentricities, 5º and 10º, in the four cardinal directions, twice at each location. The eight target locations are indicated by black Xs. Crowding thresholds are displayed as horizontal and vertical lines. Each line's length is twice the crowding distance such that the endpoints of the line indicate flanker locations at threshold spacing. Observer 2's lines are slightly offset to avoid overlap. Dashed sectors represent the part of the visual field shown in Figure 2a in the main text.

bioRxiv preprint doi: https://doi.org/10.1101/2024.04.03.587977; this version posted April 29, 2024. The copyright holder for this preprint (which was not certified by peer review) is the author/funder, who has granted bioRxiv a license to display the preprint in perpetuity. It is made available under aCC-BY 4.0 International license.

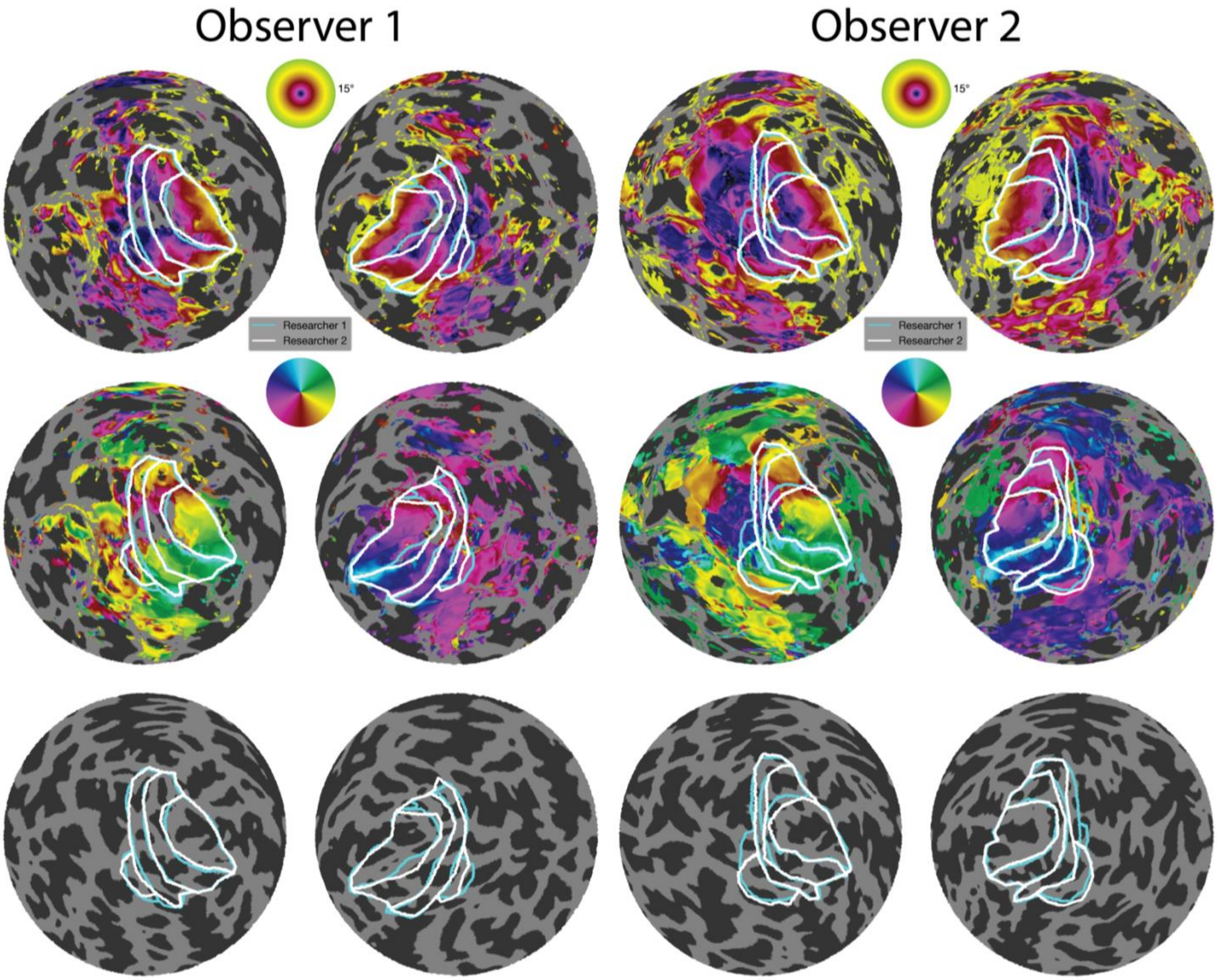

Supplementary Figure 2. Retinotopic maps for Observers 1 and 2. For each observer we show flattened representations of their left and right hemispheres. The overlays show eccentricity (top), polar angle (middle) and underlying thresholded cortical curvature (bottom; dark regions are sulci, light regions are gyri). The two sets of outlines (blue and white) are the map boundaries drawn independently by two researchers. In rows 1 and 2, the color overlays are thresholded by pRF variance explained > 10%.

bioRxiv preprint doi: https://doi.org/10.1101/2024.04.03.587977; this version posted April 29, 2024. The copyright holder for this preprint (which was not certified by peer review) is the author/funder, who has granted bioRxiv a license to display the preprint in perpetuity. It is made available under aCC-BY 4.0 International license.

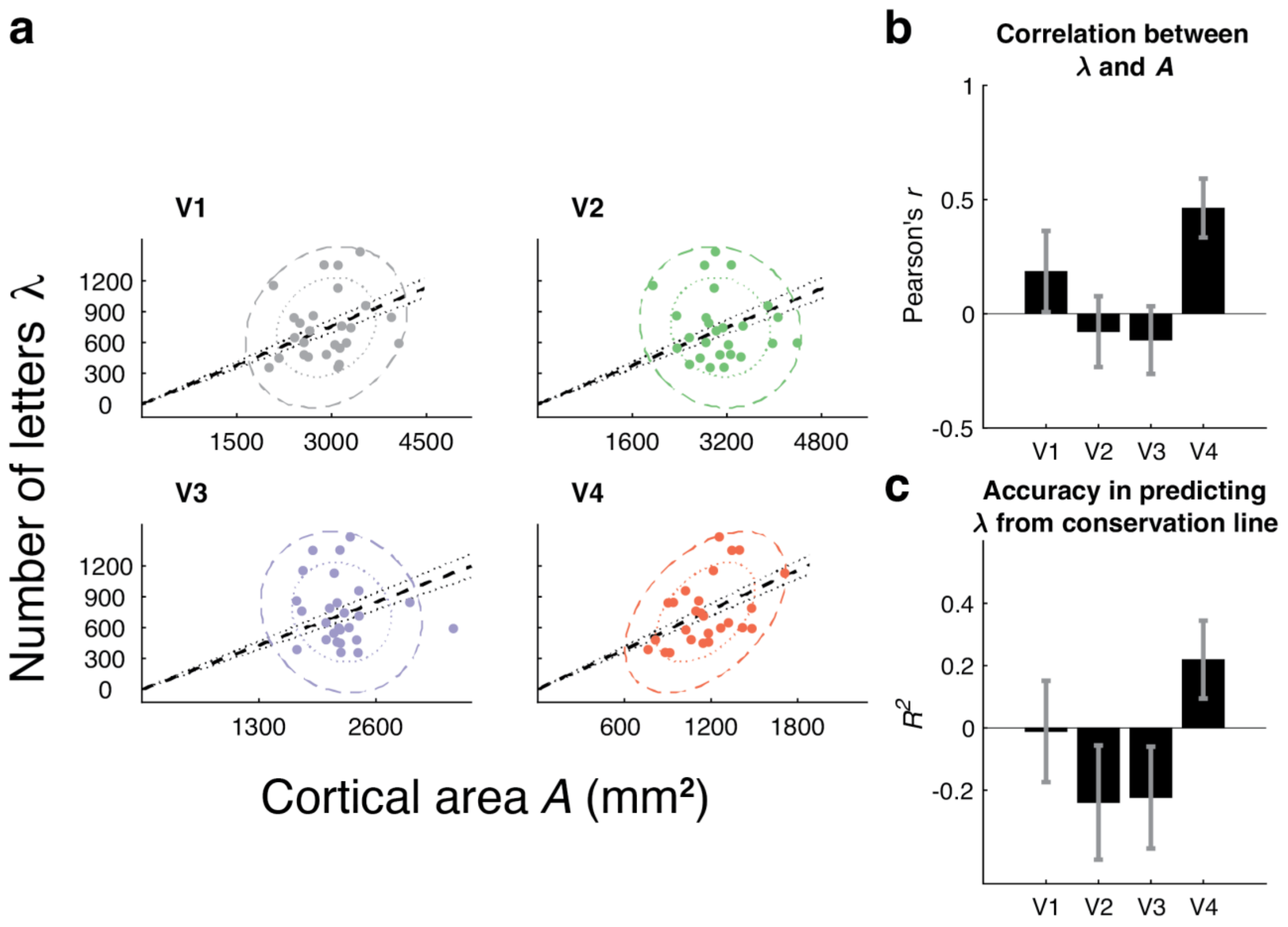

d

		Pilot	Main
Methods	Number of participants	26	49 (50 planned)
	Nature of research	Exploratory	Confirmatory
	Crowding sessions per subject	1	2
	Number of researchers defining map boundaries	1	2
	pRF stimulus design for better mapping of the fovea[1]	no	yes
Results	k (letters / mm²) in V4	0.64 [CI 0.60 - 0.69]	0.54 [CI 0.52 - 0.57]
	c (mm per letter) in V4	1.25 [CI 1.20 - 1.30]	1.36 [CI 1.33 - 1.39]
	r (crowding distance, surface area) V4	0.46	0.66
	R^2 (conservation prediction), V4	0.22 [CI 0.09 - 0.33]	0.43 [CI 0.33 - 0.50]

Supplementary Figure 3. **A comparison of pilot and main experiments a,** Number of letters λ vs cortical surface area A for the 26 observers in the pilot dataset. Otherwise plotted as in Figure 3a. **b,** The correlation between λ and *A* for V1 to V4 in the pilot dataset. Otherwise plotted as in Figure 3b **c,** Variance explained by conservation prediction. Plotted as in Figure 3c. **d**, A comparison of methods and results between the pilot and main experiments.

bioRxiv preprint doi: https://doi.org/10.1101/2024.04.03.587977; this version posted April 29, 2024. The copyright holder for this preprint (which was not certified by peer review) is the author/funder, who has granted bioRxiv a license to display the preprint in perpetuity. It is made available under aCC-BY 4.0 International license.

Supplementary Analysis 1: Sensitivity analysis of the estimated cortical crowding distance *c*
Effects of α and φ_0 on c.

We concluded that cortical crowding distance is conserved on the V4 map, meaning that there is a single value for *c* (cortical crowding distance) across participants, estimated to be 1.4 mm. We estimated this value by regressing λ, number of uncrowded letters, on *A*, the surface area of the V4 map, assuming an intercept of 0 (eq 1 in main text), and then solving for *c* (eq 3 in main text). Further, we reported uncertainty in *c* from bootstrapping across participants (**Figure 3**). Here we assess how c is affected by variation of λ across participants. The main-text calculation of λ assumed fixed values from our previous work[2] for two parameters: the tangential-to-radial ratio α of crowding distances and the Bouma law intercept φ_0. Here we calculate how the known parameter variations would affect the *c* estimate. We computed *c* 20,000 times, each time assuming different α values with φ_0 fixed (10,000 calculations), or different φ_0 values with α fixed (10,000 calculations). The values for the two parameters were based on the measured distributions, rather than assuming the mean as in the main text. From our prior study, the mean value of α was 2.10 (sd = 0.38), and the mean value of φ_0 was 0.24 (sd 0.05). Supplementary Table 1 below reports the *c* median and 95% confidence intervals from the calculations.

Median [95% CI] estimate of *c*, assuming:<br>α (μ = 2.10, $\boldsymbol{\sigma}$ = 0.38),<br>φ_0 = 0.24	Median [95% CI] estimate of c, assuming:<br>α = 2.10,<br>φ_0 (μ = 0.24, $\boldsymbol{\sigma}$ = 0.05)
1.32 [1.14,1.64]	1.36 [1.25,1.45]

Supplementary Table 1. **Sensitivity of cortical crowding distance to variation in α and φ_0.**

bioRxiv preprint doi: https://doi.org/10.1101/2024.04.03.587977; this version posted April 29, 2024. The copyright holder for this preprint (which was not certified by peer review) is the author/funder, who has granted bioRxiv a license to display the preprint in perpetuity. It is made available under aCC-BY 4.0 International license.

Supplementary Analysis 2. *Effect of $\varphi_{\max}$ on λ*.

The formula (Eq. 7) for number λ of uncrowded letters grows approximately logarithmically with $\varphi_{\max}/\varphi_0$ (**Supplementary Figure 4,** below). Our decision to measure (in psychophysics and fMRI) out to 10 deg eccentricity was somewhat arbitrary, but we can use Eq. 7 to consider how many more uncrowded letters might fit in the far periphery. As expected of logarithmic dependence, large changes in the maximum eccentricity produce only modest changes in the number of letters. For instance, doubling the maximum eccentricity $\varphi_{\max}$ from 10 to 20 deg would increase the number λ of uncrowded letters by only 13%, and increasing it tenfold, from 10 to 100 deg, would increase λ by 47%.

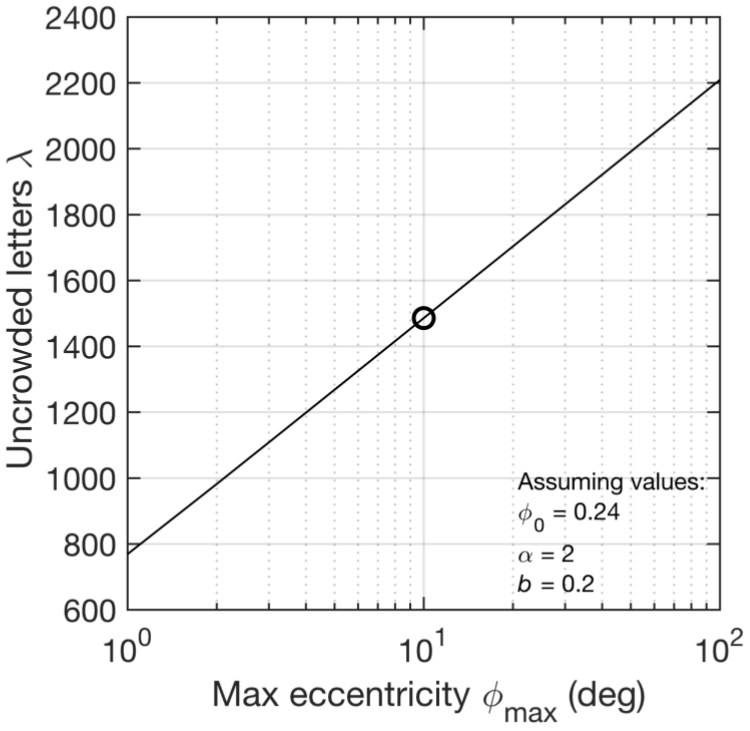

Supplementary Figure 4. **Sensitivity of number λ of uncrowded letters to maximum eccentricity $\varphi_{\max}$.** Plots the formula (Eq. 7) for λ vs. $\varphi_{\max}$. This paper mostly sets $\varphi_{\max}$ to 10 deg, shown by the circle. The nearly straight curve shows that the formula's dependence on $\varphi_{\max}$ is practically logarithmic.

bioRxiv preprint doi: https://doi.org/10.1101/2024.04.03.587977; this version posted April 29, 2024. The copyright holder for this preprint (which was not certified by peer review) is the author/funder, who has granted bioRxiv a license to display the preprint in perpetuity. It is made available under aCC-BY 4.0 International license.

Supplementary Table 1: Physiology-based links between crowding and visual brain areas

**Brain area**	**Species**	**Reference**
V1	human	Fang and He 2008[3]
V1-V3	human	Kwon et al. 2014[4]
V1-V4	human	Millin et al. 2014[5]
V1-V4	human	Anderson et al. 2012[6]
beyond V1	human	He et al. 1996[7]
V2	human	He, Wang, and Fang 2019[8]
V2	macaque, human	Freeman and Simoncelli 2011; Freeman et al. 2013[9,10]
V2-V3	human	Bi et al. 2009[11]
V2-LOC	human	Jastrzębowska et al. 2021[12]
V3	human	Tyler et al. 2007[13]
beyond V3	human	Liu et al. 2009[14]
V4	macaque	Henry and Kohn 2022[15]
V4	macaque	Motter 2018[16]

bioRxiv preprint doi: https://doi.org/10.1101/2024.04.03.587977; this version posted April 29, 2024. The copyright holder for this preprint (which was not certified by peer review) is the author/funder, who has granted bioRxiv a license to display the preprint in perpetuity. It is made available under aCC-BY 4.0 International license.

References

1 Himmelberg, M. M. *et al.* Cross-dataset reproducibility of human retinotopic maps. *NeuroImage* **244**, 118609 (2021). https://doi.org/10.1016/j.neuroimage.2021.118609

2 Kurzawski, J. W. *et al.* The Bouma law accounts for crowding in 50 observers. *Journal of Vision* **23**, 6 (2023). https://doi.org/10.1167/jov.23.8.6

3 Fang, F. & He, S. Crowding alters the spatial distribution of attention modulation in human primary visual cortex. *Journal of Vision* **8**, 6 (2008). https://doi.org/10.1167/8.9.6

4 Kwon, M., Bao, P., Millin, R. & Tjan, B. S. Radial-tangential anisotropy of crowding in the early visual areas. *Journal of Neurophysiology* **112**, 2413-2422 (2014). https://doi.org/10.1152/jn.00476.2014

5 Millin, R., Arman, A. C., Chung, S. T. L. & Tjan, B. S. Visual Crowding in V1. *Cereb Cortex* **24**, 3107-3115 (2014). https://doi.org/10.1093/cercor/bht159

6 Anderson, E. J., Dakin, S. C., Schwarzkopf, D. S., Rees, G. & Greenwood, J. A. The neural correlates of crowding-induced changes in appearance. *Curr Biol* **22**, 1199-1206 (2012). https://doi.org/10.1016/j.cub.2012.04.063

7 He, S., Cavanagh, P. & Intriligator, J. Attentional resolution and the locus of visual awareness. *Nature* **383**, 334-337 (1996). https://doi.org/10.1038/383334a0

8 He, D., Wang, Y. & Fang, F. The Critical Role of V2 Population Receptive Fields in Visual Orientation Crowding. *Curr Biol* **29**, 2229-2236.e2223 (2019). https://doi.org/10.1016/j.cub.2019.05.068

9 Freeman, J. & Simoncelli, E. P. Metamers of the ventral stream. *Nat. Neurosci.* **14**, 1195-1201 (2011). https://doi.org/10.1038/nn.2889

10 Freeman, J., Ziemba, C. M., Heeger, D. J., Simoncelli, E. P. & Movshon, J. A. A functional and perceptual signature of the second visual area in primates. *Nat Neurosci* **16**, 974-981 (2013). https://doi.org/10.1038/nn.3402

11 Bi, T., Cai, P., Zhou, T. & Fang, F. The effect of crowding on orientation-selective adaptation in human early visual cortex. *Journal of Vision* **9**, 13.11-10 (2009). https://doi.org/10.1167/9.11.13

12 Jastrzębowska, M. A., Chicherov, V., Draganski, B. & Herzog, M. H. Unraveling brain interactions in vision: The example of crowding. *NeuroImage* **240**, 118390 (2021). https://doi.org/10.1016/j.neuroimage.2021.118390

13 Tyler, C. W. & Likova, L. T. Crowding: a neuroanalytic approach. *J Vis* **7**, 16 11-19 (2007). https://doi.org/10.1167/7.2.16

14 Liu, T., Jiang, Y., Sun, X. & He, S. Reduction of the crowding effect in spatially adjacent but cortically remote visual stimuli. *Curr Biol* **19**, 127-132 (2009). https://doi.org/10.1016/j.cub.2008.11.065

15 Henry, C. A. & Kohn, A. Feature representation under crowding in macaque V1 and V4 neuronal populations. *Curr Biol* **32**, 5126-5137.e5123 (2022). https://doi.org/10.1016/j.cub.2022.10.049

16 Motter, B. C. Stimulus conflation and tuning selectivity in V4 neurons: a model of visual crowding. *Journal of Vision* **18**, 15 (2018). https://doi.org/10.1167/18.1.15
