# Supplementary material for: Conservation of cortical crowding distance across individuals in human V4
Source: bioRxiv. 2024 Apr 5:2024.04.03.587977. Preprint. [Version 1] doi: 10.1101/2024.04.03.587977 (PMC11014589; doi:10.1101/2024.04.03.587977)
Supplement: Supplementary file 1 [file NIHPP2024.04.03.587977V1-supplement-1.pdf]

bioRxiv preprint doi: https://doi.org/10.1101/2024.04.03.587977; this version posted April 5, 2024. The copyright holder for this preprint (which was not certified by peer review) is the author/funder, who has granted bioRxiv a license to display the preprint in perpetuity. It is made available under aCC-BY 4.0 International license.

Supplementary materials

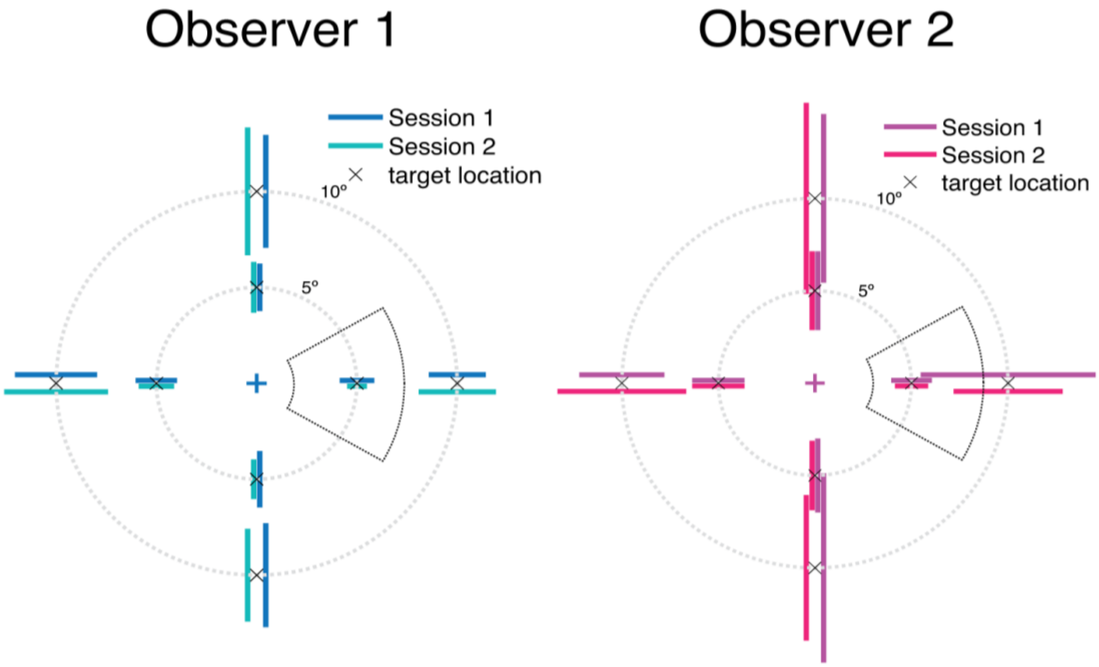

Supplementary Figure 1. Crowding distances for Observers 1 and 2. The crowding distance was measured radially at two eccentricities, 5º and 10º, and the four cardinal directions, twice at each location. Target locations are the black Xs. Each line's length is twice the crowding distance such that the endpoints of the line indicate flanker locations. For observer 2, the lines were slightly jittered to avoid overlap. Dotted sectors represent the part of the visual field shown in Figure 2A in the main text.

bioRxiv preprint doi: https://doi.org/10.1101/2024.04.03.587977; this version posted April 5, 2024. The copyright holder for this preprint (which was not certified by peer review) is the author/funder, who has granted bioRxiv a license to display the preprint in perpetuity. It is made available under aCC-BY 4.0 International license.

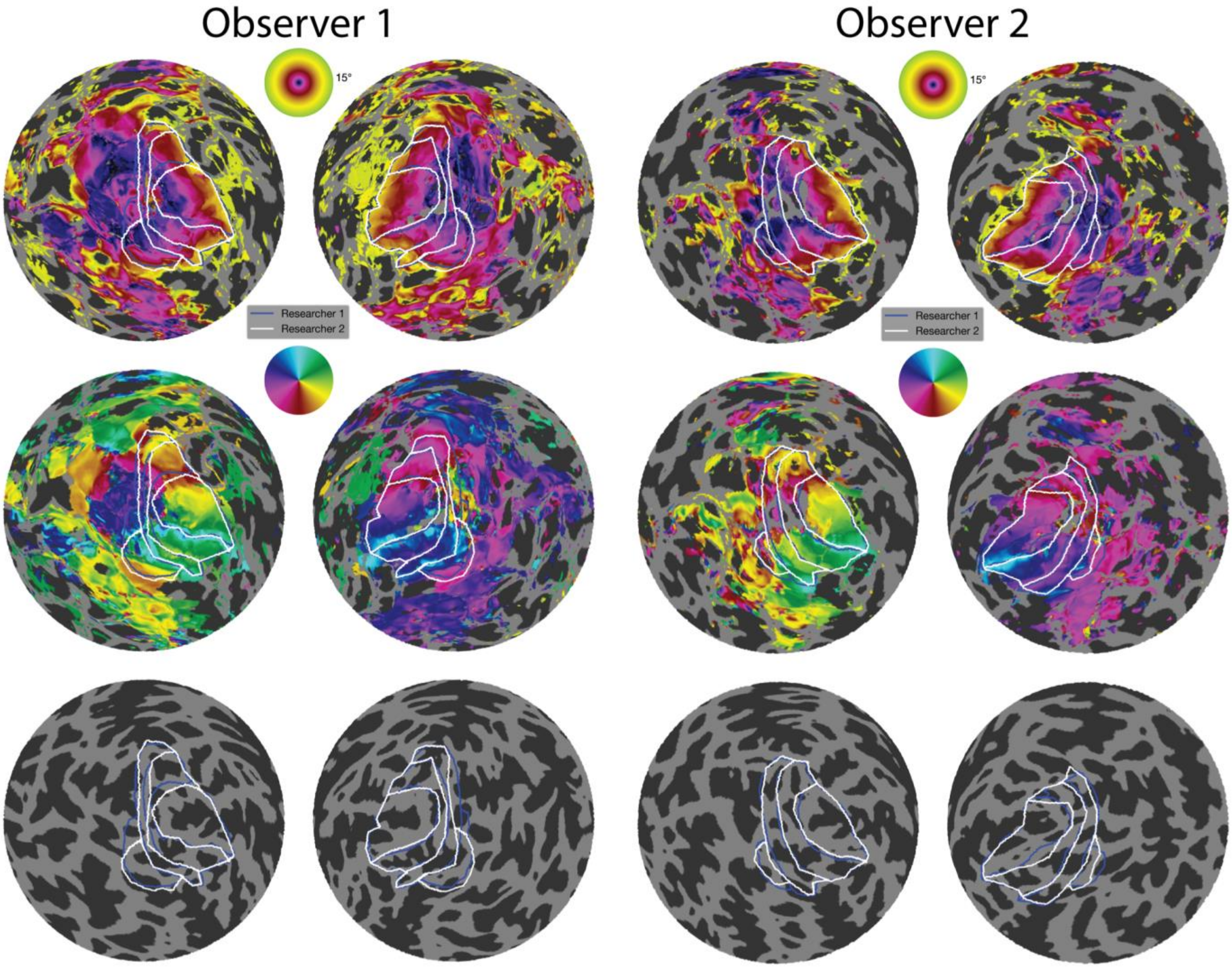

Supplementary Figure 2. Retinotopic maps for Observers 1 and 2. For each observer we show flattened representations of their left and right hemispheres. The overlays show eccentricity (top), polar angle (middle) and underlying thresholded cortical curvature (bottom; dark regions are sulci, light regions are gyri). The two sets of outlines (blue and white) are the map boundaries drawn independently by two researchers. In rows 1 and 2, the color overlays are thresholded by pRF variance explained > 10%.

bioRxiv preprint doi: https://doi.org/10.1101/2024.04.03.587977; this version posted April 5, 2024. The copyright holder for this preprint (which was not certified by peer review) is the author/funder, who has granted bioRxiv a license to display the preprint in perpetuity. It is made available under aCC-BY 4.0 International license.

Supplementary analysis 1: Sensitivity analysis of the estimated c
Our conclusion was that cortical crowding distance is conserved on the V4 map, meaning that there is a single value for *c* (cortical crowding distance) across participants, which we estimated as 1.4 mm. We estimated this value by linear regression: number of letters in the uncrowded visual field, λ, vs surface area of the V4 map, *A*, for which we have 49 paired measures:

$\hat{\lambda} = kA$, where $\hat{\lambda}$ is the estimate of λ

and

$$c = \frac{1}{\sqrt{k}}$$

In the main text, we report how participant sampling affects *c* (**Figure 3**). Here we test how uncertainty in λ affects our estimate of c. To derive λ we assumed values for the ratio between tangential and radial crowding distance, α, and the intercept of the Bouma law, φ_0, based on our previous work[1]. Here we calculate how these assumptions influence the estimate of *c*. We computed *c* 20,000 times, each time assuming different α values and with fixed φ_0 (10,000 calculations), or different values of φ_0 with fixed α (10,000 calculations). The values for the two parameters were based on the measured distributions, rather than assuming the mean as in the main text. From our prior study, the mean value of α was 2.10 (std = 0.38), and the mean value of φ_0 was 0.24 (std 0.05). We report the values of *c* (median and 95% confidence intervals) from the calculations as follows:

Estimated values of *c* assuming variation in α and φ_0	α (μ = 2.10, $\boldsymbol{\sigma}$ = 0.38),<br>φ_0=0.24	α = 2.10,<br>φ_0 (μ = 0.24, $\boldsymbol{\sigma}$ = 0.05)
c	1.32 [1.14-1.64]<br>(median [95% CI])	1.36 [1.25-1.45]<br>(median [95% CI])

bioRxiv preprint doi: https://doi.org/10.1101/2024.04.03.587977; this version posted April 5, 2024. The copyright holder for this preprint (which was not certified by peer review) is the author/funder, who has granted bioRxiv a license to display the preprint in perpetuity. It is made available under aCC-BY 4.0 International license.

Supplementary Table 1: Physiology-based links between crowding and visual brain area

Brain area	Species	Reference
V1	human	(Fang and He 2008)[2]
V1-V3	human	(Kwon et al. 2014)[3]
V1-V4	human	(Millin et al. 2014)[4]
V1-V4	human	(Anderson et al. 2012)[5]
V2	human	(He, Wang, and Fang 2019)[6]
V2	macaque, human	(Freeman and Simoncelli 2011; Freeman et al. 2013)[7,8]
V2-V3	human	(Bi et al. 2009)[9]
V2-LOC	human	(Jastrzębowska et al. 2021)[10]
V3	human	(Tyler et al. 2007)[11]
beyond V3	human	(Liu et al. 2009)[12]
V4	macaque	(Henry and Kohn 2022)[13]
V4	macaque	(Motter 2018)[14]

bioRxiv preprint doi: https://doi.org/10.1101/2024.04.03.587977; this version posted April 5, 2024. The copyright holder for this preprint (which was not certified by peer review) is the author/funder, who has granted bioRxiv a license to display the preprint in perpetuity. It is made available under aCC-BY 4.0 International license.

References

1 Kurzawski, J. W. *et al.* The Bouma law accounts for crowding in 50 observers. *Journal of Vision* **23**, 6, doi:10.1167/jov.23.8.6 (2023).

2 Fang, F. & He, S. Crowding alters the spatial distribution of attention modulation in human primary visual cortex. *Journal of Vision* **8**, 6, doi:10.1167/8.9.6 (2008).

3 Kwon, M., Bao, P., Millin, R. & Tjan, B. S. Radial-tangential anisotropy of crowding in the early visual areas. *Journal of Neurophysiology* **112**, 2413-2422, doi:10.1152/jn.00476.2014 (2014).

4 Millin, R., Arman, A. C., Chung, S. T. L. & Tjan, B. S. Visual Crowding in V1. *Cereb Cortex* **24**, 3107-3115, doi:10.1093/cercor/bht159 (2014).

5 Anderson, E. J., Dakin, S. C., Schwarzkopf, D. S., Rees, G. & Greenwood, J. A. The neural correlates of crowding-induced changes in appearance. *Curr Biol* **22**, 1199-1206, doi:10.1016/j.cub.2012.04.063 (2012).

6 He, D., Wang, Y. & Fang, F. The Critical Role of V2 Population Receptive Fields in Visual Orientation Crowding. *Curr Biol* **29**, 2229-2236.e2223, doi:10.1016/j.cub.2019.05.068 (2019).

7 Freeman, J. & Simoncelli, E. P. Metamers of the ventral stream. *Nat. Neurosci.* **14**, 1195-1201, doi:10.1038/nn.2889 (2011).

8 Freeman, J., Ziemba, C. M., Heeger, D. J., Simoncelli, E. P. & Movshon, J. A. A functional and perceptual signature of the second visual area in primates. *Nat Neurosci* **16**, 974-981, doi:10.1038/nn.3402 (2013).

9 Bi, T., Cai, P., Zhou, T. & Fang, F. The effect of crowding on orientation-selective adaptation in human early visual cortex. *Journal of Vision* **9**, 13.11-10, doi:10.1167/9.11.13 (2009).

10 Jastrzębowska, M. A., Chicherov, V., Draganski, B. & Herzog, M. H. Unraveling brain interactions in vision: The example of crowding. *NeuroImage* **240**, 118390, doi:10.1016/j.neuroimage.2021.118390 (2021).

11 Tyler, C. W. & Likova, L. T. Crowding: a neuroanalytic approach. *J Vis* **7**, 16 11-19, doi:10.1167/7.2.16 (2007).

12 Liu, T., Jiang, Y., Sun, X. & He, S. Reduction of the crowding effect in spatially adjacent but cortically remote visual stimuli. *Curr Biol* **19**, 127-132, doi:10.1016/j.cub.2008.11.065 (2009).

13 Henry, C. A. & Kohn, A. Feature representation under crowding in macaque V1 and V4 neuronal populations. *Curr Biol* **32**, 5126-5137.e5123, doi:10.1016/j.cub.2022.10.049 (2022).

14 Motter, B. C. Stimulus conflation and tuning selectivity in V4 neurons: a model of visual crowding. *Journal of Vision* **18**, 15, doi:10.1167/18.1.15 (2018).
